# Expression profiling of the Dof gene family under abiotic stresses in spinach

**DOI:** 10.1038/s41598-021-93383-6

**Published:** 2021-07-13

**Authors:** Hongying Yu, Yaying Ma, Yijing Lu, Jingjing Yue, Ray Ming

**Affiliations:** 1 College of Agriculture, Center for Genomics and Biotechnology, Fujian Provincial Key Laboratory of Haixia Applied Plant Systems Biology, Fujian Agriculture and Forestry University, Fuzhou, 350002 Fujian China; 2 Center for Genomics and Biotechnology, Fujian Agriculture and Forestry University, Fuzhou, 350002 Fujian China; 3 Department of Plant Biology, University of Illinois at Urbana-Champaign, Urbana, IL 61801 USA

**Keywords:** Genetics, Plant genetics

## Abstract

DNA-binding with one finger (Dof) are plant-specific transcription factors involved in numerous pathways of plant development, such as abiotic stresses responses. Although genome-wide analysis of *Dof* genes has been performed in many species, but these genes in spinach have not been analyzed yet. We performed a genome-wide analysis and characterization of *Dof* gene family in spinach (*Spinacia oleracea* L.). Twenty-two *Dof* genes were identified and classified into four groups with nine subgroups, which was further corroborated by gene structure and motif analyses. Ka/Ks analysis revealed that *SoDofs* were subjected to purifying selection. Using *cis*-acting elements analysis, *SoDofs* were involved in plant growth and development, plant hormones, and stress responses. Expression profiling demonstrated that *SoDofs* expressed in leaf and inflorescence, and responded to cold, heat, and drought stresses. *SoDof22* expressed the highest level in male flowers and under cold stress. These results provided a genome-wide analysis of *SoDof* genes, their gender- and tissue-specific expression, and response to abiotic stresses. The knowledge and resources gained from these analyses will benefit spinach improvement.

## Introduction

Spinach (*Spinacia oleracea* L.) is an annual or biennial diploid species, belong to the Amaranthaceae family in the order Caryophyllales^1^ Its annual worldwide gross production in 2016 was about 26 million tonnes (FAOSTAT; http://faostat3.fao.org). Spinach is a dietary source of Ca, Cu, Fe, K, Mg, Mn, P, Zn, folate, vitamins, and dietary fiber^2^, providing its great potential for medical economy^3,4^. However, like many other crops, its development and production is hampered by biotic stresses(diseases, pests and weed infestations,) and abiotic stresses (salinity, drought, and heat)^5^. Climate change causes elevated temperature and a network of events triggering the response of plants and animals^6,7^. Although it seems that organisms on earth gradually developed local thermal adaptation to impact their healthy condition^8^. Spinach is cold tolerant but having heat-sensitive characteristics that influencing its growth and significantly decrease yield and quality under hight temperature^9^. Winter sweet treatment (WST), termed the cold enrichment technique, has been established for cultivating high-quality leafy spinach during winter^10^. At that time (early December), the average daily temperature is generally below 5 °C. But staying at a low temperature for a long time would also damage spinach by reactive oxygen species (ROS)^11^. Although drought stress has no direct effects on the leaf nutrition quality, some physiological indicators could be decreased, such as leaf area, fresh and dry weight, leaf relative water content, and specific leaf area, which might change the shape of plant^12^.

Dof domain proteins are plant-specific transcription factors that contain a highly conserved 52 amino acid DNA-binding domain at the N-terminalincluding a single Cys2/Cys2 zinc finger structure^13^. It was projected that Cys2/Cys2 zinc finger specifically binds to a conserved sequence with 5′-(T/A)AAAG-3′ in gene promoters^14^. At the C-terminal of the Dof proteins, there is a transcription regulation domain with diverse functions involving interaction with a variety of regulatory proteins and activating the gene expression^15^. Indeed, previous studies corroborated its functional role in plant growth and development, such as in flowering control^16,17^, maturation^18^, seed development^19^, and germination^20,21^. Specifically, mutant dag1 (encoding a *Dof* transcription factor in Arabidopsis) seeds are induced to germinate by much lower red light fluence rates^22^; the *COG1* gene (encoding a Dof protein in Arabidopsis) functions as a negative regulator in phytochrome signaling pathways^23^; *CDFs* (CYCLING DOF FACTORS, Dof-type transcriptional repressors) that directly suppresses the expression of CONSTANS (*CO*), which could prevent the expression of photoperiodic gene, the perception of day-length and the floral transition in Arabidopsis^24^. Moreover, *Dof* transcription factors also participated in phytohormone and stress responses, such as the *TDDF1* (encoding a Dof protein in tomato) which could improve drought, salt, various hormones stress as well as resistance to late blight^25^; *ThZFP1* and *ThDof1.4* improve salt and osmotic stress tolerance by increase the proline level and ROS scavenging capability^26^. Therefore, Dof gene family plays an essential role in the life cycle of plants.

In recent years, with the sequencing of genome, the identification of Dof genes was widely researched in various plant species, such as *Arabidopsis*, rice^27^, soybean^28^, maize^29^, sorghum^30^, sugarcane^31^, and so on. The spinach draft genome was reported in 2017^1^, however, few gene families were analyzed for the genome. The functions of members of *Dof* genes remain unknown in spinach. As previously reported, plants different sex types show different responses to abiotic stress^32^. The reproductive potential of male, female, and monoecious spinach differe under water-limited condition^33^. But the expression of Dof genes in different sex types of spinach under abiotic stresses is still unknown. In this study, we identified 22 *Dof* genes, showed the structure and motifs, and classified the group of *Dof* genes in spinach. In addition, duplication events and *cis*-element on their promoters were predicted. Functional prediction was performed based on gene expression analysis in different tissues and in responses to different abiotic stresses. The results will provide a foundation for gene cloning and functional characterization of *Dofs* in spinach.

## Materials and methods

### Identification of *SoDof* gene family members in the spinach genome

To identify the *Dof* gene family members in *Spinacia oleracea* L., all proteins from the spinach genome were scanned by HMMER-3.2^34^ using the Hidden Markov Model (HMM) corresponding to the HMM profile of the Dof domain (PF02701). The spinach genome data was downloaded from SpinachBase (http://www.spinachbase.org/?q=download). The predicted proteins were confirmed for the presence of the conserved Dof domain by NCBI Conserved Domain Database (CDD)^35^, Pfam^36^ and SMART^37^ tools. Similarly, Arabidopsis and sugarbeet (*Beta vulgaris* L.) *Dof* genes were identified by scanning Arabidopsis database (ftp://ftp.ensemblgenomes.org/pub/plants/release-42/fasta/arabidopsis_thaliana/) and sugarbeet database (ftp://ftp.ensemblgenomes.org/pub/plants/release-42/fasta/beta_vulgaris/) using HMM and CDD. We performed the ExPASy server^38^ to detect the theoretical pI and molecular weight of candidate *SoDof* genes.

### Multiple sequences alignment and phylogenetic characterization

For phylogenetic analysis of the Dof gene family, multiple sequence alignments were conducted on the amino acid sequences of Dof protein from spinach, Arabidopsis, and sugarbeet by MUSCLE with default settings. After that, MEGA-X-10.0.4 software was used to construct phylogenetic tree among these three species with the Neighbour-Joining (NJ) method and 1000 bootstraps. Alignment of multiple *SoDofs* was performed by DNAMAN-6.0.

### Chromosomal locations and duplication time

The distribution information for each *SoDof* gene on chromosome was obtained from their annotation file. MG2C (http://mg2c.iask.in/mg2c_v2.1/) was used to map the chromosomal locations for each *SoDof* gene with default settings. To estimate the synonymous and non-synonymous substitution, Ka and Ks values were calculated. ClustalW was used to align the nucleotide sequence of *SoDof* genes. Ka and Ks values were used to estimate by DnaSp-5.10. The time (million years ago, Mya) of segmental duplication events for each *SoDof* gene was estimated using a formula, T = Ks/2λ which assumed λ of 7.0e^−9^ synonymous/substitution site/year for spinach^1^.

### Gene structure analysis and conserved motif identification

The exon–intron organizations of the genes with phylogenetic tree and Dof motifs were determined using the Gene Structure Display Server (http://gsds.cbi.pku.edu.cn/). The motifs distribution of the Dof protein in spinach, Arabidopsis, and sugarbeet were statistically identified by the MEME program (http://meme-suite.org/) with the motif length set to 6–100 and the maximum number of motifs was set to 15. Then TBtools-1.082^39^ was employed to create the motif structure with phylogenetic tree.

### *Cis*-elements identification in promoter regions of SoDofs

To investigate *cis*-elements in promoter sequences of *Dof* coding genes in spinach, the upstream sequences (2000 bp) of each *SoDof* gene were extracted from spinach genome according to the GFF3 (general feature format) file. Then the retrieved sequences were submitted to a search by the PlantCARE (http://bioinformatics.psb.ugent.be/webtools/plantcare/html/)^40^ for predicting the *cis*-elements which might be involved in regulation of *SoDof* genes expression.

### Sample collection and preparation

Spinach II9A0073 seeds were obtained from CAAS (China Academy of Agricultural Sciences). Seeds were sown in plots, and seedlings grew in an artificial climate chamber with a photoperiod of 16 h light/8 h dark, temperature at 24 °C and humidity at about 60%. After three weeks, spinach seedlings with consistent growth were selected and prepared for environmental stress treatment. Abiotic stresses were performed by adding 20% (mass fraction) PEG 4000 to simulate the drought condition and adjusting the temperature of the artificial climate box to simulate high-temperature stress (40 °C) and low-temperature stress (4 °C). Under stress conditions, the spinach leaves were sampled at 0, 2, 4, 7, 12, 24 h after treatment. The plants with non-treatment were collected for their roots, leaves, and stems in vegetative growth stage, as well as their male flowers and female flowers. All samples were immediately frozen in liquid nitrogen and stored at − 80 °C.

### RNA extraction and quantitative real-time PCR analysis

Total RNA from different samples was extracted using the Trizol reagent. The quality and concentration of RNA were tested on 1.0% agar gel electrophoresis and the NanoDrop 2000 (Thermo Fisher Scientific, USA). The total RNA was reverse transcribed into cDNA with its 200 ng per microliter final work concentration using Evo M-MLV RT Kit with gDNA Clean for qPCR (Accurate Biotechnology, China) according to the manufacturer’s instruction. For qRT-PCR, *Actin11* gene was used as a reference gene. The specific primers were designed by IDT (https://sg.idtdna.com/pages) and the sequences of all primers are listed in Supplementary Table S3. The qRT-PCR was conducted with SYBR Green Premix Pro Taq HS qPCR Kit (Accurate Biotechnology, China) following the manufacturer’s protocol. Experiments were repeated three times with technical and biological replications for each sample. The relative gene expression level was calculated by the 2 − ∆∆CT method. Graphpad Prism8 (Graphpad Software Inc., La Jolla, CA) was performed to calculate the *p*-value. Expression values were calculated as the arithmetic mean and then presented as the heatmap by R package.

## Result

### Identification and classification of SoDofs genes

To identify the *Dof* gene family members in spinach, all proteins from the spinach genome were scanned by using HMMER-3.2 and 22 genes were predicted as *Dof* gene family members in spinach. These *Dof* candidate genes in spinach were named as *SoDof*1–*SoDof*22 (Table 1). The predicted proteins were further confirmed to contain the conserved Dof domain. Similarly, 36 *Dof* genes had been identified in Arabidopsis and 22 *Dof* genes were identified in sugarbeet named as *BvDof1*–*BvDof22* (Supplementary Table S1). The full length of the coding sequence (CDS) ranged from 492 (*SoDof12*) bp to 1485 (*SoDof13*) bp with an average length of 1060 bp. The quantity of aa (amino acids) for *SoDof* varied from 163 (*SoDof12*) to 494 (*SoDof13*) aa, with an average protein length of ~ 352 aa. The molecular weight (MW) fluctuated between 18.5 kDa (*SoDof12*) and 54.5 kDa (*SoDof13*), and the theoretical isoelectric points (pI) ranged from 4.6 (*SoDof20*) to 8.92 (*SoDof9*) (Table 1).

**Table 1:** Spinach Dof genes and their related information. Forward means that the gene is located on the negative stand of chromosome; reverse means the gene is located on the positive stand of chromosome.

Gene name	Gene ID	Chromosome	Location	Gene DNA (bp)	CDS (bp)	Protein length (aa)	Molecular weight	Theoretical pI	Dof domain	Intron	Subgroup
*SoDof1*	Spo01218	chr2	58115820..58118612 forward	2793	1104	367	40,642.53	8.52	57–114	1	C2.1
*SoDof2*	Spo26525	chr4	115910084..115910743 reverse	660	660	219	23,339.72	8.47	23–79	0	A
*SoDof3*	Spo14528	chr3	51468026..51469123 forward	1098	1098	365	39,514.46	7.32	41–96	0	B2
*SoDof4*	Spo15329	chr5	13015823..13016842 forward	1020	1020	339	37,310.74	5.59	52–108	0	A
*SoDof5*	Spo26037	chr6	40210301..40212930 forward	2630	1197	398	44,408.07	6.25	58–115	1	C2.1
*SoDof6*	Spo25524	SpoScf_02134	33891..35945 reverse	2055	1287	428	46,606.00	8.80	90–146	1	B2
*SoDof7*	Spo19252	chr5	6739988..6741368 reverse	1381	1110	369	39,234.09	6.93	47–104	1	C1
*SoDof8*	Spo19232	SpoScf_01574	110099..110860 reverse	762	762	253	25,482.25	8.12	28–83	0	D2
*SoDof9*	Spo13986	SpoScf_01503	63276..64439 reverse	1164	1165	387	41,004.88	8.92	79–135	0	B2
*SoDof10*	Spo20892	Super_scaffold_114	1245494..1248131 reverse	2638	1326	441	46,968.23	8.21	95–150	1	B1
*SoDof11*	Spo08108	chr5	10912882..10916291 forward	3410	1344	447	49,445.56	5.39	108–164	1	D1
*SoDof12*	Spo04353	SpoScf_01506	92311..92802 forward	492	492	163	18,468.93	8.87	44–99	0	D1
*SoDof13*	Spo05430	SpoScf_01199	340472..345369 forward	4898	1485	494	54,499.48	5.63	154–210	1	D1
*SoDof14*	Spo16539	SpoScf_00408	13249..16754 forward	3506	1059	352	38,506.78	6.46	99–155	1	D1
*SoDof15*	Spo26832	chr6	26503975..26505054 reverse	1080	1080	359	40,449.97	6.23	28–82	0	C2.2
*SoDof16*	Spo22565	chr1	19149992..19151942 reverse	1951	1098	365	39,747.75	8.50	84–138	1	B1
*SoDof17*	Spo22229	SpoScf_01420	149590..151164 forward	1575	1101	366	40,015.00	8.51	87–141	1	B1
*SoDof18*	Spo07164	SpoScf_08285	1203..2777 forward	1575	1101	366	40,027.05	8.51	87–141	1	B1
*SoDof19*	Spo25703	Super_scaffold_205	553984..554928 reverse	945	945	314	35,306.63	8.53	58–111	0	B2
*SoDof20*	Spo00332	chr4	83899644..83900468 reverse	825	825	274	30,538.30	4.60	34–88	0	C2.2
*SoDof21*	Spo10686	chr1	41630415..41632583 forward	2169	1305	434	47,592.39	5.74	149–205	1	D1
*SoDof22*	Spo16511	SpoScf_00982	142499..143254 forward	756	756	251	27,368.16	7.60	44–98	0	C3

**Figure 1:**
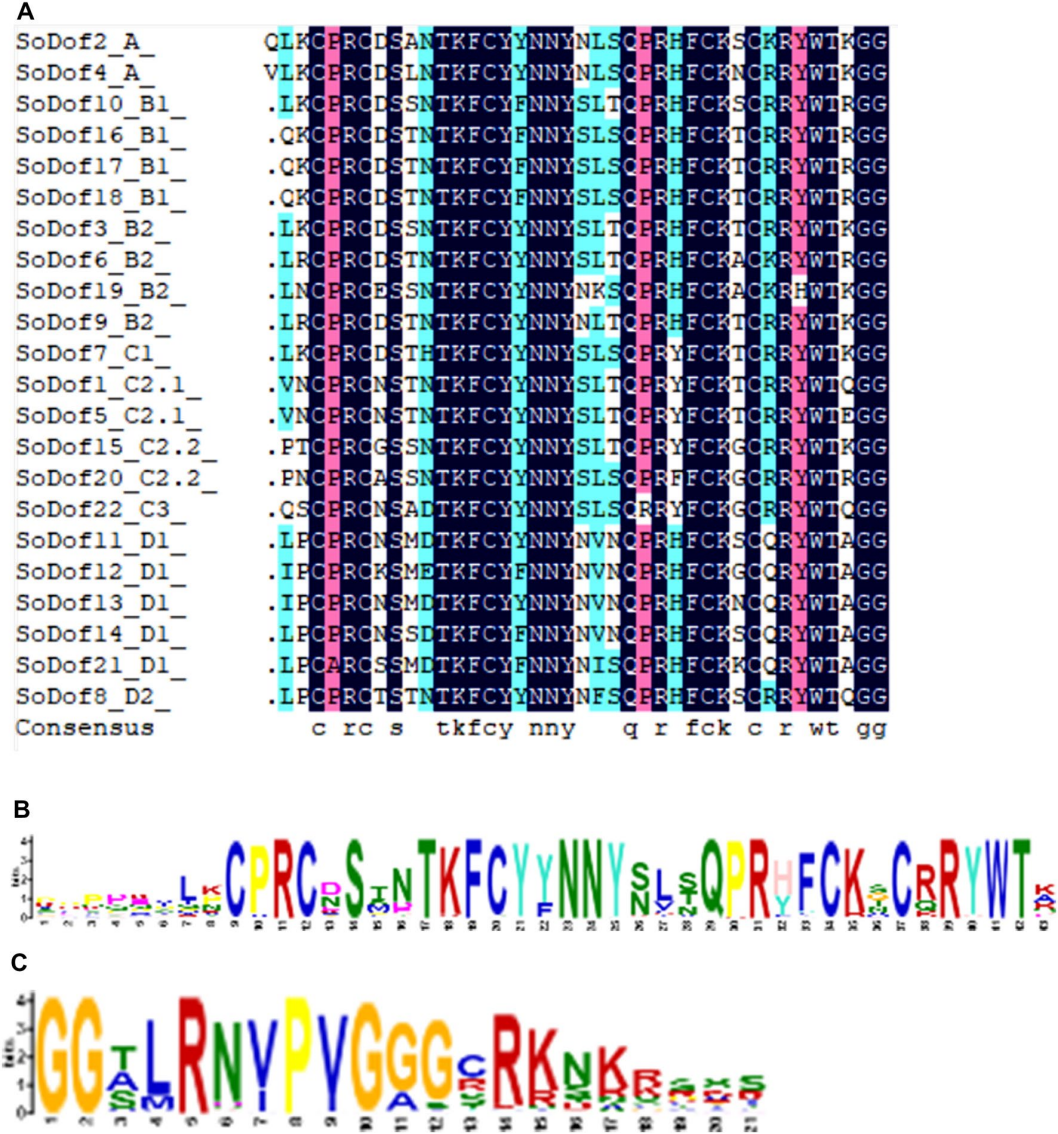
The Dof concerved region in *SoDofs*. (**A**) Alignment of multiple protein sequences in SoDofs. (**B**) Conserved amino acid sequences of motif1 by MEME. (**C**) Conserved amino acid sequences of motif2 by MEME. Figure (**A**) was made by DNAMAN-6.0.

**Figure 2:**
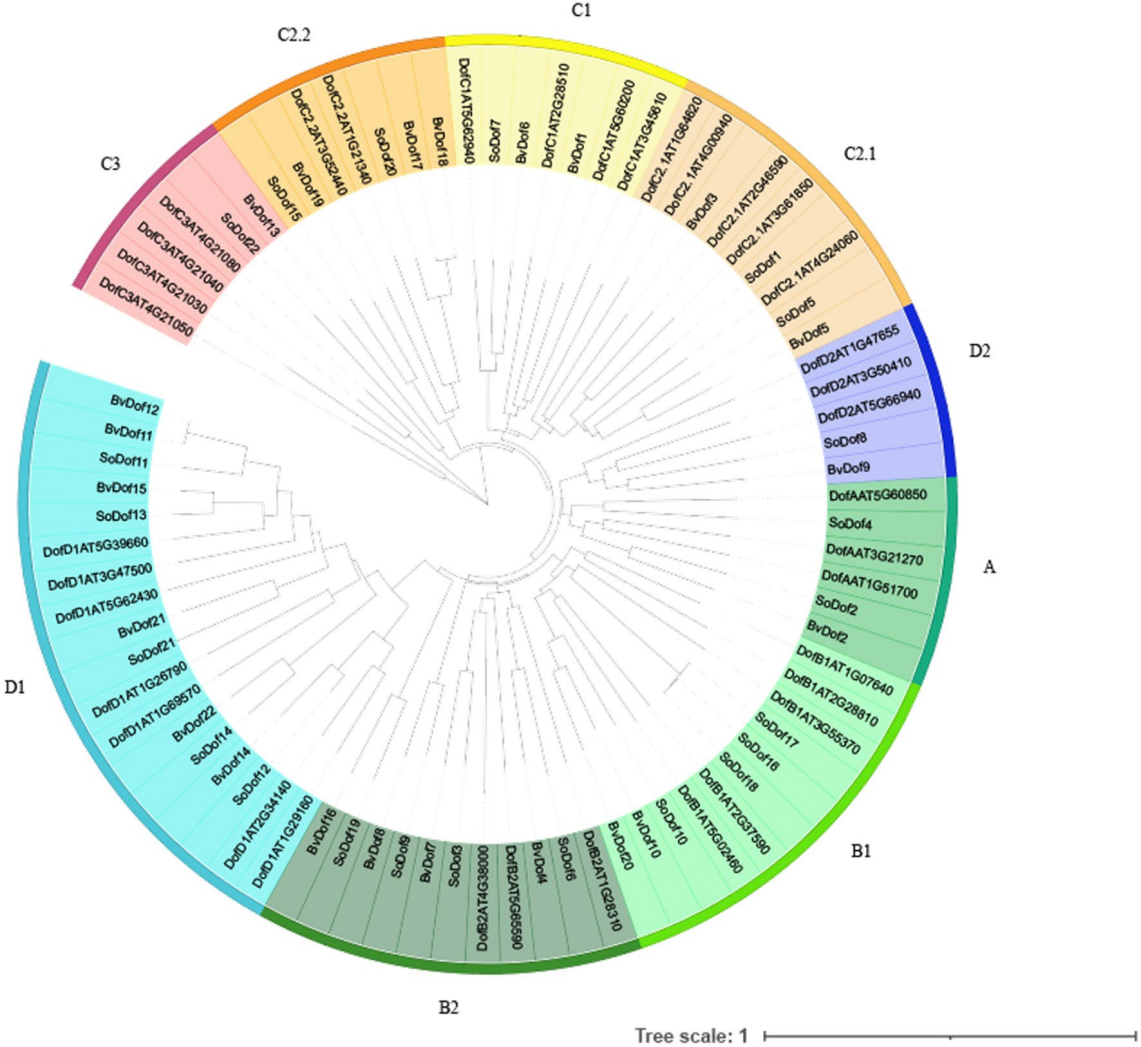
Phylogenetic tree of *Dof* proteins among spinach, *Arabidopsis* and sugarbeet. Figure was made by MEGA-X-10.0.4.

Multiple sequence alignment showed a Dof conserved motif of 52 amino acids located in 22 *SoDof* genes, with a single Cys2/Cys2 zinc-finger structure at the N-terminal (Fig. 1A). Phylogenetic tree was constructed between 22 *SoDof* genes, 22 *BvDof* genes, and 36 *Dofs* in Arabidopsis (Fig. 2). A total of 22 *SoDof* TFs from spinach were classified into four main groups (Groups A–D), which could be divided into multiple subgroups, A, B1, B2, C1, C2.1, C2.2, C3, D1, and D2. The number of *SoDofs* in Group B, C, and D was similar with a total number of 20. Specifically, Group B (contained the most number among all groups) could be divided into subgroup B1 and subgroup B2 with *SoDof10*, *SoDof16*, *SoDof17*, *SoDof18* in subgroup B1 and *SoDof3*, *SoDof6, SoDof9, SoDof19* in subgroup B2 (Fig. 2). Subgroup D1 had the largest number of *SoDofs* (*SoDof11*, *SoDof12*, *SoDof13*, *SoDof14*, *SoDof21*) in subgroups. *SoDof2* and *SoDof4* belonged to Group A (Fig. 2). Over half *SoDofs* were alkaline which contained all members in Group B, and subgroup D1 (Table 1).

### Mapping *SoDof* genes in spinach chromosomes and Ka/Ks analysis

**Figure 3:**
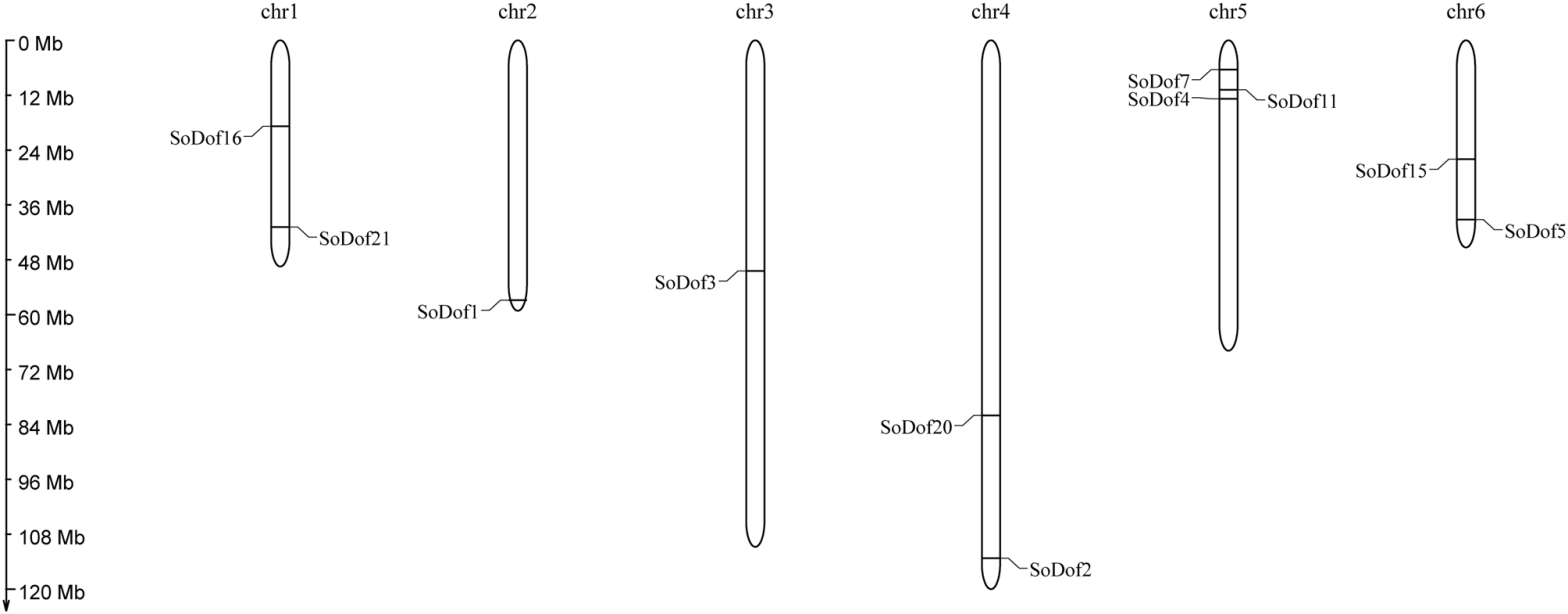
Chromsomal location of *SoDof* genes. The size of a chromosome is indicated by its relative length. Figure was made by MG2C (http://mg2c.iask.in/mg2c_v2.1/).

The spinach genome consists of only 6 chromosomes. The 22 putative *SoDof* genes were found to be distributed in 6 chromosomes, and unplaced contigs (Fig. 3). Only 50% *SoDofs* genes were anchored in chromosomes. The largest number of *SoDof* members was located in chromosome 5, which contains *SoDof* 7*,* 11, and 4. Compared with the gap of *SoDof* in other chromosomes, these three genes were closer to each other, especially *SoDof11* and *SoDof4*. There were 2 *SoDof* genes in chromosomes 1, 4, and 6, respectively. *SoDof1* and *SoDof3* were located in chromosomes 2 and 3, respectively. Ka and Ks value calculation aims to identify duplication events for each *SoDof* gene. The duplication of *SoDof* genes originated from about 5.66 Mya (Ks = 0.793) to 41.27 Mya (Ks = 5.778) with an average of 16.12 Mya (Supplementary Table S2). All values of Ka/Ks were lower than 1 and some *SoDof* were even lower than 0.1 (Table 2).

**Table 2:** The Ka/Ks value of SoDof genes (lower than 0.1). The details Ka/Ks information are shown in Supplementary Table S2.

Seq1	Seq2	Ks	Ka	Time (mya)	Ka/Ks
SpoDof2	SpoDof3	5.2612	0.3741	37.58	0.071105451
SpoDof4	SpoDof7	5.2531	0.5222	37.52214286	0.099407969
SpoDof5	SpoDof15	4.1515	0.3321	29.65357143	0.079995182
SpoDof12	SpoDof21	3.7472	0.2989	26.76571429	0.079766225
SpoDof20	SpoDof22	5.7779	0.4813	11.68785714	0.083300161

### Gene structure and motif analysis of SoDof genes

**Figure 4:**
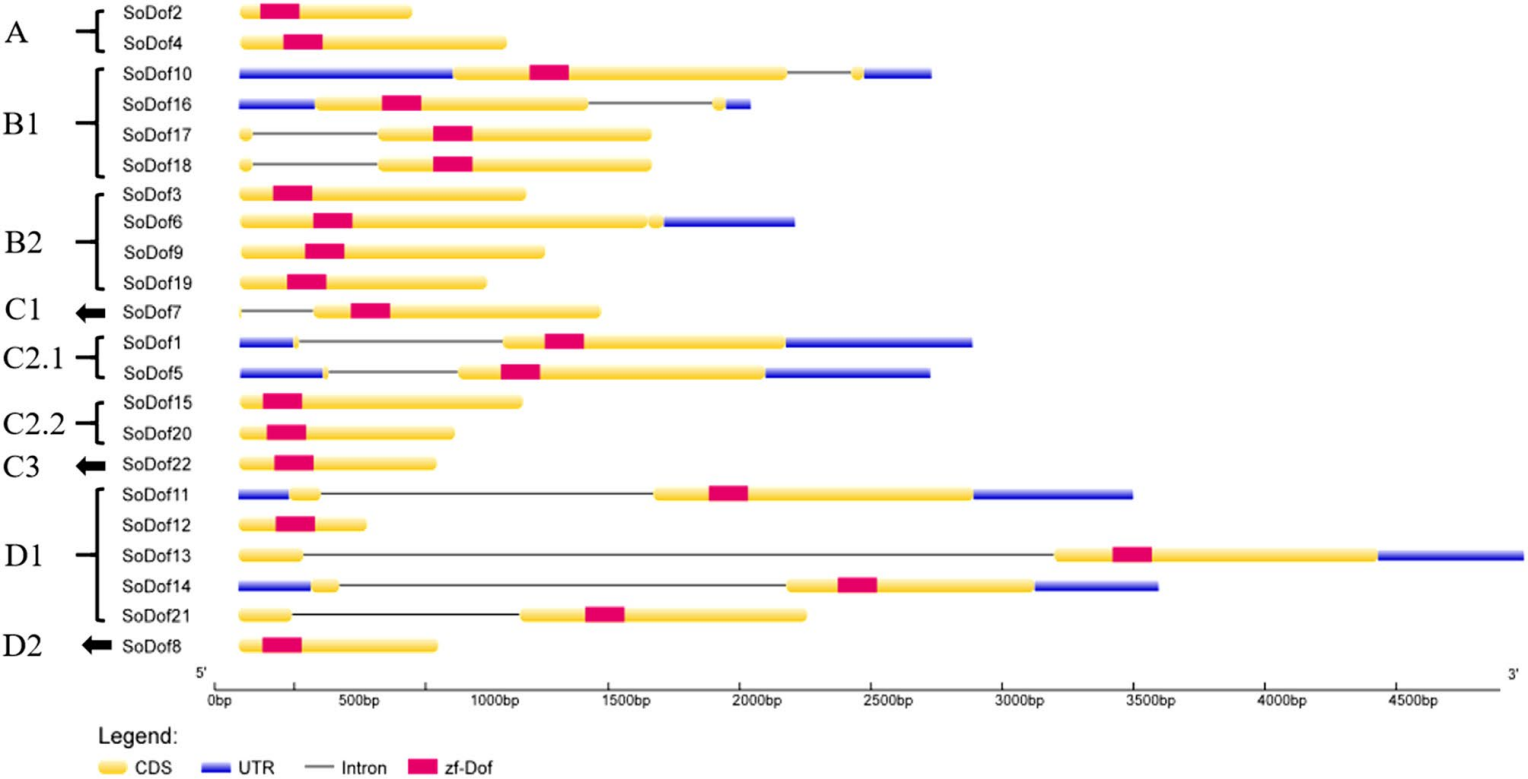
The exon–intron structure of Dof genes in Spinach: CDS (yellow), UTR (blue), Intron (black line) and zf-Dof region (pink). *SoDof6* contains one intron which is too short to recognize in this figure resolution. Figure was made by the Gene Structure Display Server (http://gsds.cbi.pku.edu.cn/).

**Figure 5:**
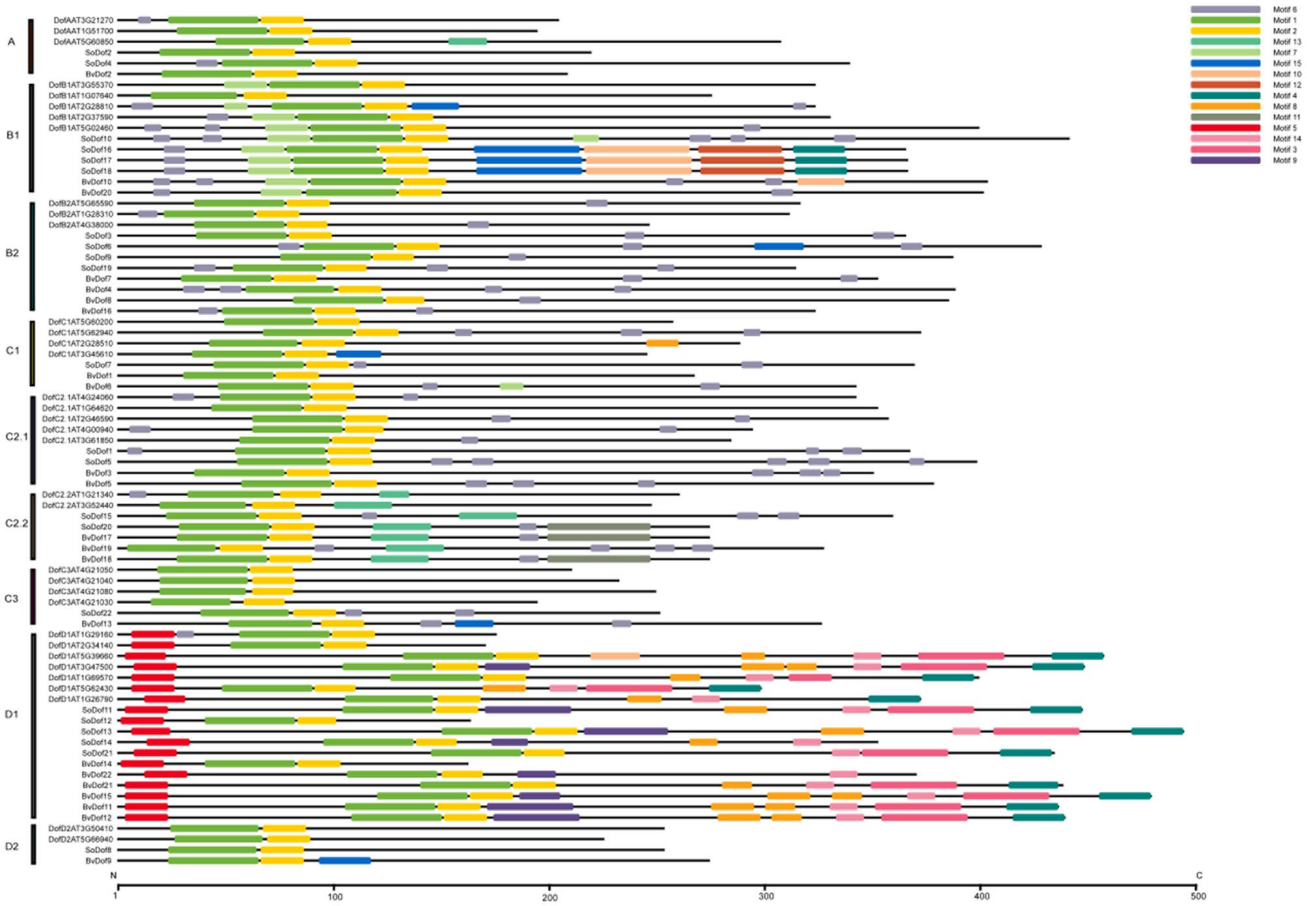
The schematic distribution of motifs for *Dof* genes among spinach, *Arabidopsis* and sugarbeet. Figure was made by the MEME program (http://meme-suite.org/) and TBtools-1.082.

Candidate *SoDof* genes were analyzed using Gene Structure Display Server to investigate the characterization of exon–intron structure. There was no more than two introns in each *SoDof* (Fig. 4). To further reveal the diversification of *SoDof* genes, we performed the MEME program to detect motif patterns, and 15 distinct motifs were identified (Fig. 5). It was predicted that motif1 could be considered as the Dof region (Fig. 1B). The schematic distribution of the 15 motifs showed that motif1 (Fig. 1B) and motif2 (Fig. 1C) were highly conserved in all *SoDof* proteins. Notably, *SoDofs* shared similar conserved motif compositions in some subgroups. Motif 7 in front of the Dof region were highly conserved in subgroup B1. And members of subgroup C2.2 contained motif13. Interestingly, motif5 was prominently conserved in subgroup D1 (contained the most *SoDof* members among all subgroups). Specifically, motif5 presented at the N-terminal in all subgroup D1 members, and motif4 appeared at the C-terminal in majority of subgroup D1 members.

### Cis-regulatory element analysis

PlantCARE was used to analyze the *cis*-regulatory element for each *SoDof* gene by retrieving the 2 kb upstream sequence of each candidate, except for *SoDof18* because of lack of 2 kb upstream sequence on its scaffold location (Supplementary Data). Dof gene family in spinach had TATA-box and CAAT-box. *SoDof* genes may also be controlled by many phytohormones, such as methyl jasmonate (MeJA), gibberellins (GA), ethylene, auxin, and salicylic acid (SA). We also detected many other important *cis*-elements on Dof gene family that involve in plant growth and development. For example, there were a large number of elements associated with physiological processes, such as light responsiveness, circadian control, endosperm expression, meristem and flower meristem expression, root-specific and seed-specific regulation (Supplementary Data). The sum of *cis*-elements of subgroup D1 was greatest in plant growth and development. The sum of *cis*-elements of subgroup D1 was also greatest in phytohormones class. The greatest mean of *cis*-elements in phytohormones class was subgroup C3. The greatest mean of *cis*-elements in light responsiveness and physiological process were in subgroup C2.2 and C1 respectively (Table 3). In physiological process, some elements, participated in some small molecule pathway, were also found, such as zein metabolism regulation and flavonoid biosynthetic genes regulation (Supplementary Data). Moreover, nine *cis*-elements (WUN-motif, STRE, TC-rich repeats e.g.) were also predicted, which were related to defense and stress responsiveness. The sum and mean of *cis*-elements of subgroup A were greatest in stress response.

**Table 3:** The sum and mean of *cis*-elements for each subgroup.

Subgroup	Growth and development				Phytohormone response		Stress response	
	Light responsiveness		Physiological pathways					
	Sum	Mean	Sum	Mean	Sum	Mean	Sum	Mean
A	33	16.5	19	9.5	33	16.5	49	24.5
B1	56	18.67	6	2	38	12.67	34	11.33
B2	86	21.5	19	4.75	63	15.75	49	12.25
C1	19	19	13	13	10	10	7	7
C2.1	43	21.5	16	8	35	17.5	14	14
C2.2	52	26	9	4.5	32	16	13	13
C3	14	14	4	4	30	30	5	5
D1	99	19.8	33	6.6	96	19.2	43	8.6
D2	17	17	6	6	23	23	15	15

### Tissue-specific expression analysis of *SoDof* genes

**Figure 6:**
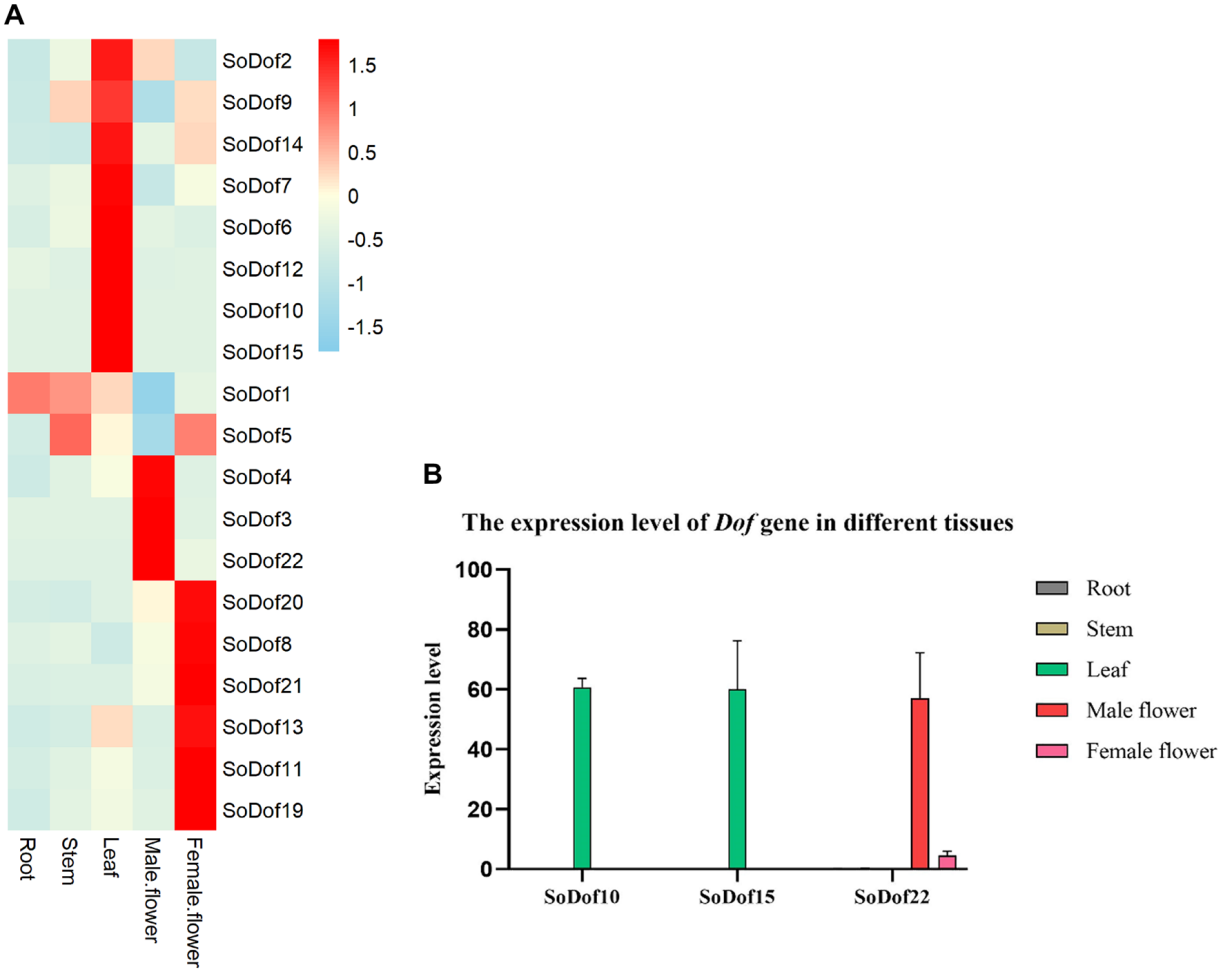
The tissue-specific expression of *Dof* genes in Spinach by qRT-PCR. (**A**) Expression level of *SoDofs*. The color scheme used to present expression level is sky-blue/red: light-yellow boxes indicate low variation in gene expression, sky-blue indicate a fold decrease, and red boxes indicate a fold increase in relation to mean value. The expression value were calculated as the arithmetic mean. (**B**) The expression level of *SoDof10*, *SoDof15* and *SoDof22* in different tissues. The Y-axis indicates relative expression level and the X-axis indicated different tissues: root (gray); stem (light brown); leaf (green); female flower (red); male flower (pink). The error bars were caculated based on three biological repiticates using standard deviation. Figure (**A**) was made by R package; (**B**) was made by Graphpad Prism8.

We isolated RNA samples from roots, stems, leaves, male flowers, and female flowers, and detected expression of all *SoDof* genes in spinach using qRT-PCR. Expression profile of the *SoDof* genes revealed that nine *SoDofs*exhibited their highest transcript level in reproductive organs and eight *SoDofs* in leaves (Fig. 6A). Only two *SoDofs* (*SoDof1* and *SoDof5*) were expressed in roots and stems, respectively. Notably, *SoDof10* and *SoDof15* had extremely high expression in leaves; *SoDof22* showed high expression in male flowers (Fig. 6B). Comparing with leaves or inflorescences, the transcript level of these three genes in other tissues was neglectable, indicating that their expression was tissue-specific. There were three homologous genes (*SoDof16*, *SoDof17,* and *SoDof18*) with same mRNA sequence, and their expression pattern was not analyzed.

### Expression patterns of *SoDof* genes under abiotic stresses

To investigate the stress responsiveness and expression pattern of *SoDof* gene between different sex-types, we treated female male plants, and plants at vegetative stage under three types of abiotic stress (low-temperature 4 °C, high-temperature 40 °C, and drought 20%PEG4000). Spinach leaves were collected at 0 h, 2 h, 4 h, 7 h, 12 h, and 24 h after treatment and detected by qRT-PCR.

**Figure 7:**
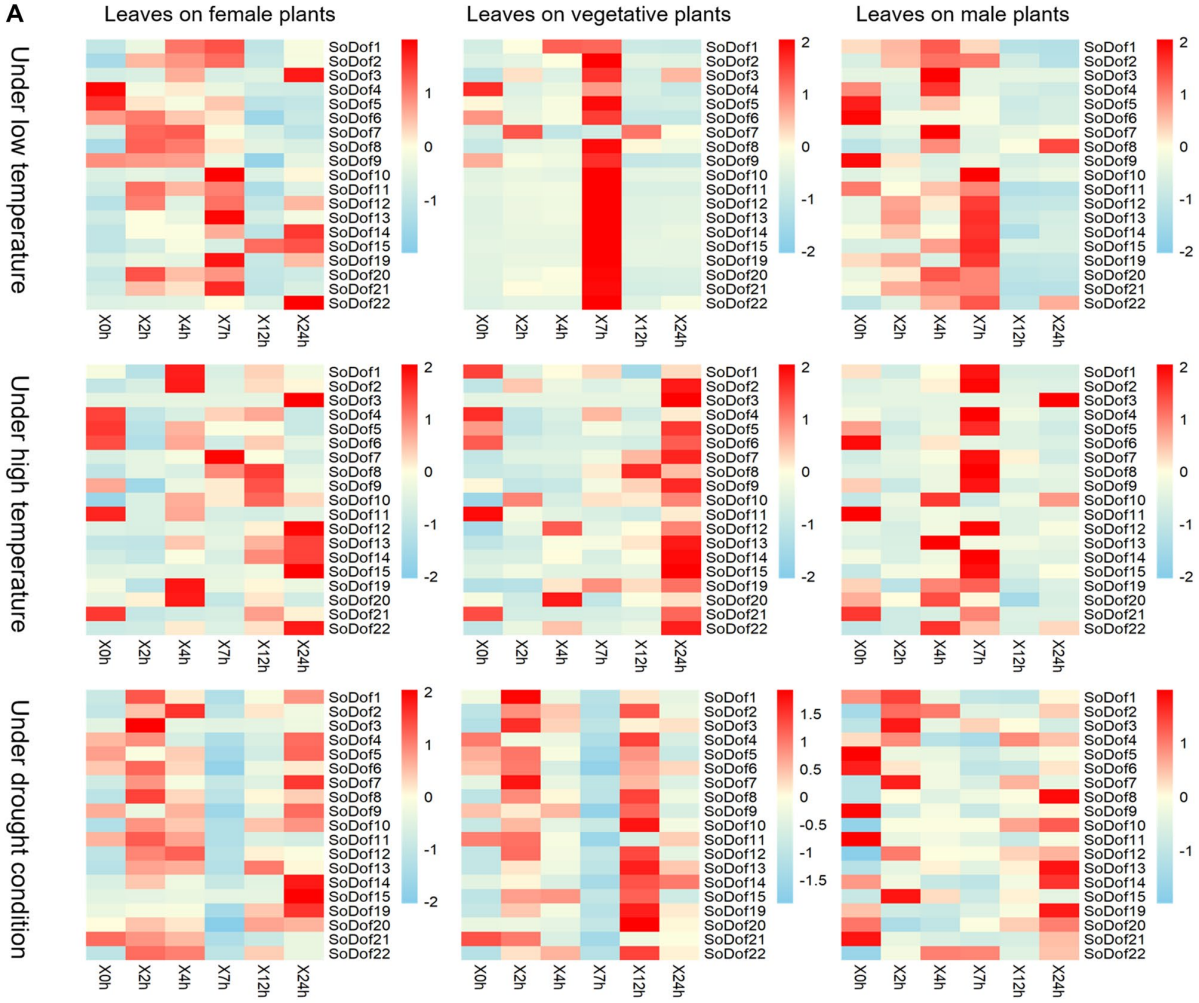

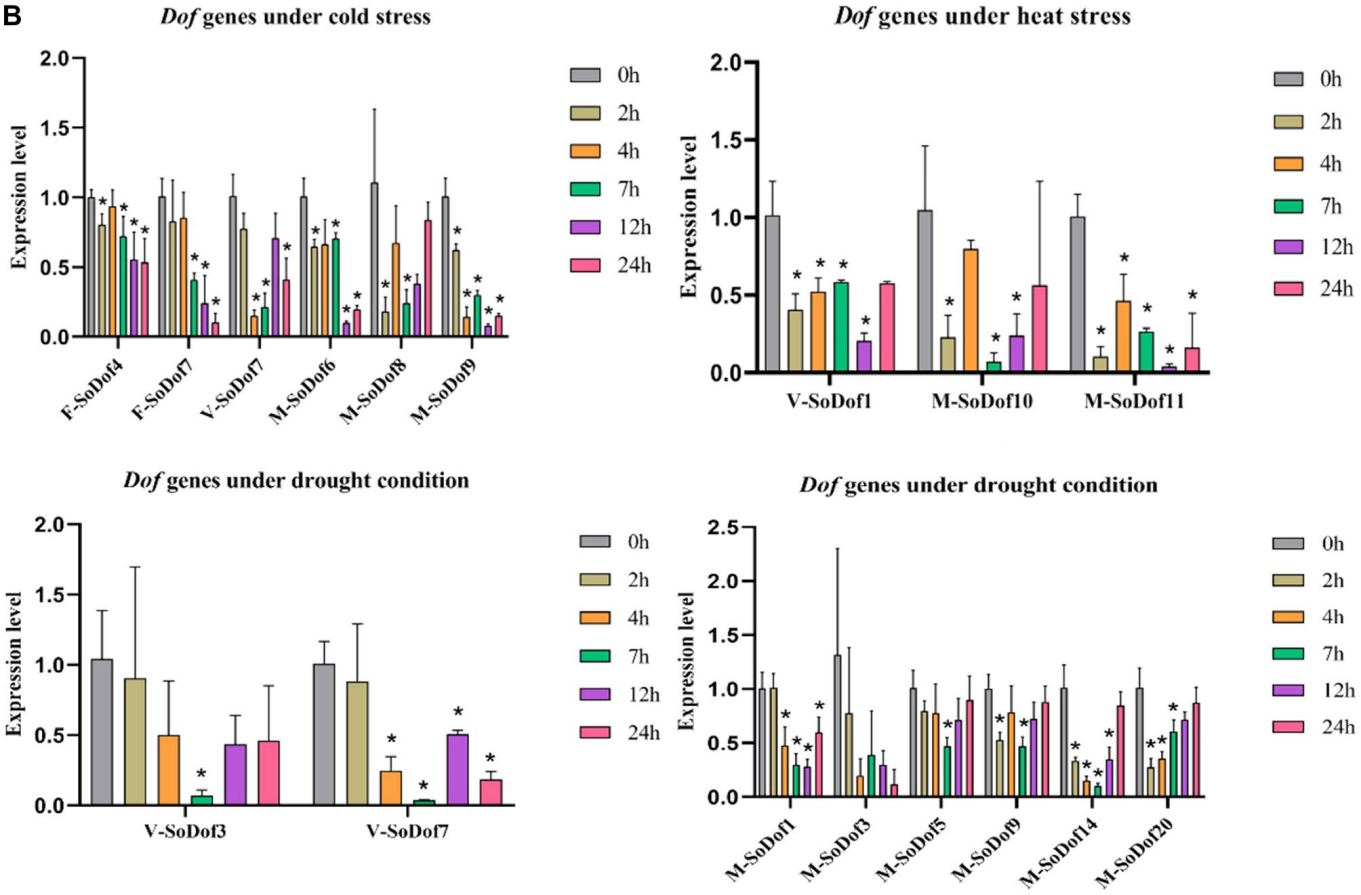
The expression pattern of *SoDof* genes under stresses. (**A**) The expression pattern of all *SoDof* genes under cold stress, heat stress and drought stress. The color scheme used to present expression level is sky-blue/red: light-yellow boxes indicate low variation in gene expression, sky-blue indicate a fold decrease, and red boxes indicate a fold increase in relation to mean value. The Y-axis indicates each *SoDof* gene and the X-axis indicated the time after treatment. The expression value were calculated as the arithmetic mean. (**B**) The expression level of down-regulated *SoDofs*. F-*SoDof* means the *SoDof* gene in female plants; V-*SoDof* means the *SoDof* gene in vegetative plants; M-*SoDof* means the *SoDof* gene in male plants. The Y-axis indicates relative expression level and the X-axis indicated the time after treatment:0 h (gray); 2 h (light brown); 4 h (orange); 7 h (green); 12 h (purple);24 h (pink). Asterisk indicates a significant difference from 0 h (p < 0.05). Error bars indicate standard error of independent technological replicates. Figure (**A**) and (**B**) were made by Graphpad Prism8.

The majority of *SoDof* genes in female plants were up regulated under low temperature (Fig. 7A). The greatest increase in expression occurred in *SoDof*22 (up to the top at 24 h after treatment) in female plants (Supplementary Fig. S2A). *SoDof14* experienced the same trend, but the expression level was much lower than that in *SoDof*22. Compared with other *SoDofs*, the *SoDof*22 expressed the most in plants at vegetative stage, and its extreme expression reached the top at 7 h and then went down (Supplementary Fig. S2B). However, in male plants, the expression pattern of *SoDof3* and *SoDof5* was similar. The expression of *SoDof3* reached the highest level at 4 h and the expression of *SoDof5* reached the highest level at 7 h (Supplementary Fig. S2C). In vegetative plants, 95% *SoDof* genes (more than those in male or female plants) were up-regulated and almost all of their highest expression appeared at 7 h (Fig. 7A). Among them, *SoDof3*, *SoDof4*. *SoDof8* and *SoDof9* were down-regulated at 2 h and 4 h. After that, they expressed the highest level at 7 h and then went down. The trends of six *SoDofs* (*SoDof11*, *SoDof12, SoDof13, SoDof19, SoDof20,* and *SoDof21*) were similar. Their expression went up slightly at 2 h and 4 h and reached the highest at 7 h, and then went down (Supplementary Fig. S2B). But there were difference between female and male plants. In male plants, there were the most number of *SoDofs* (*SoDof6*, *SoDof8,* and *SoDof*9) down-regulated, indicating that *SoDof* genes in males showed more negative response under 4 °C (Fig. 7B).

Under high temperature, most *SoDofs* were up-regulated and all *SoDof* genes were up-regulated in female plants. Compared with other *SoDof* genes, the expression of *SoDof3* (up to the top at 24 h) was the highest in females, males, and vegetative plants (Supplementary Fig. S3). *SoDof12*, *SoDof13*, *SoDof14*, *SoDof15,* and *SoDof22* also exhibited the highest expression at 24 h in female plants. The expression of some genes (*SoDof1*, *SoDof2*, *SoDof5*, *SoDof6*, *SoDof11*, *SoDof19,* and *SoDof20*) went up to the highest at 4 h which means they responded earlier than others did. In plants at vegetative stage, there was only one down-regulated *SoDof* gene (*SoDof*1) (Fig. 7B). Additionally, the expression of *SoDof*6, *SoDof8,* and *SoDof9* were suppressed in male plants (Fig. 7B). 68% *SoDofs* showed the highest transcript level at 24 h in plant at vegetative stage, and 84% *SoDofs* showed the highest transcript level at 7 h or before 7 h in male plants (Supplementary Fig. S3).

To investigate the expression profile for each *SoDofs* under drought condition.All *SoDof* genes were up-regulated in female plants. Compared to other *SoDof* genes, the expression of *SoDof15* was highest in females, males, and vegetative plants (Supplementary Fig. S4). But it was up to the top at 24 h in females, at 12 h in vegetative plants, and at 2 h in males. *SoDof3* and *SoDof7* were down-regulated in plants at vegetative stage (Fig. 7B). In male plants, six *SoDof* genes (*SoDof1*, *SoDof3*, *SoDof5*, *SoDof9 SoDof14,* and *SoDof20*) exhibited suppressed expression, and the expression of all *SoDofs* was lower than in female and vegetative plants (Supplementary Fig. S4).

## Discussion

### Identification and characteristics of *SoDof* genes

The *Dof* gene family is a plant-specific family of transcription factors. Since the discovery of the first *Dof* gene in maize^41^, its members in other species have been uncovered and its function in the growth and development has been characterized. We identified 22 *SoDof* genes in spinach genome and constructed a phylogenetic tree to divide them into four categories (A, B, C, and D) (Fig. 2). The quantity of *SoDofs* is lower than that of *Arabidopsis* (36)^27^, tomato (34)^42^, wheat (96)^43^, rice (30)^27^, potato (35)^44^, soybean (78)^28^, and sugarcane (29)^31^, but it is same to that of sugarbeet. This is because spinach separated with *Arabidopsis* just after the ancient whole-genome triplication and there was no whole-genome duplication in spinach genome^1^. The theoretical isoelectric points (pI) of Dof proteins ranged from 4.6 to 8.92. Only two Dof proteins have an isoelectric point between 6.5 and 7.5, and over half Dof proteins were alkaline. All values of Ka/Ks were lower than 1 (Supplementary Table S2), indicating that *SoDof* genes were subjected to purifying selection^45^.

### Structural conservation and chromosome location of *SoDof* genes

From our analysis of the spinach genome^1^, only half of the *Dof* genes were assembled in chromosomes. Their distribution was relatively even, but three *Dof* genes clustered on one end of the chromosome 5 (Fig. 3). Although the spinach genome has no recent whole-genome duplication, partial gene duplications may lead to the formation of specific *Dof* genes clustered in specific parts of chromosomes. It is the main effect on gene family expansion^46^. The exon–intron divergence is supporting evidence to determine the evolutionary relationship of plants^47^. The intron–exon analysis showed that there were no more than two introns in each *Dof* gene (Fig. 4). The distribution of motifs is indicative of an evolutionary relationship^43^. The protein sequence analysis of the 80 *Dof* genes (22 *SoDof*, 22 *BvDof*, and 36 *Dof* in Arabidopsis) revealed that only Dof motifs of these 80 protein sequences are conserved (Fig. 5). The Dof proteins in the same subgroup contain relatively conserved motif structures. Motif 7 is in subgroup B1 and motif13 is in subgroup C2.2. Motif5 were prominently conserved in the subgroup D1. Specifically, motif5,, motif3, and motif14 are only conserved in subgroup D1.

### *Cis*-elements of *SoDof* genes

*Cis*-elements play significant roles during the life cycle of plants, such as phytohormone and stress response. In *SoDof* gene family, most *cis*-elements we identified were those related to light response, revealing that light signals may influence the regulation of *SoDofs* expression. Moreover, we identified *cis*-elements associated with the development of plant tissues in the promoter region of *SoDofs*, such as AP-1^48^. *Cis*-elements associated with hormones and stress response were also identified in the promoter region of *SoDofs*. These results suggested that *SoDof* genes may participate in plant development and response to hormone and stress.

### Potential Role of *SoDof* genes in different tissues

To figure out the potential roles of *SoDofs*, we analyzed the expression profiles of 19 *SoDof* genes in different spinach tissues. The other three genes, *SoDof16*, *SoDof17,* and *SoDof18*, were excluded from the analyses because they shared the mRNA sequences that are not distinguishable from each other*.* Among the 19 *SoDofs* expressed in spinach, 42% *SoDofs* showed a dominant expression in leaves and 47% in reproductive organs (Fig. 6A). In grapevine, eleven of twenty-five *Dof* gene expressed in inflorescences^49^ (similar to the number of *SoDofs*). Over half of *Dof* genes were expressed in vascular system in spinach, as in *Arabidopsis*^50^. Among them, there are six *SoDofs* (*SoDof*4, *SoDof*11, *SoDof*19, *SoDof*20, *SoDof*21, and *SoDof*22) that expressed at a high level in flowers, indicating that they might be involved in the development of reproductive organs, especially for *SoDof*22 (Fig. 6B). *SoDof*22 is orthologous to *AT4G21050*, which is involved in regenerated shoot numbers^51^. Comparing with the number of *cis*-elements of *SoDofs*, *SoDof22* contained the most *cis*-elements associated with plant hormone. One-third of them were ERE^52^ which are ethylene-responsive elements. This gene also contained the most auxin-responsive *cis*-elements, such as AuxRR-core^53^ and TGA-box^54^. These *Dof* genes might involve in the growth and development of spinach reproductive organs.

### Potential role of *SoDof* genes in response to abiotic stress

In the expression profile for abiotic stress, the expression of *SoDofs* in male plants was lower than that in female plants and the plants at vegetative stage (Supplementary Figs. S2–S4). The trend of expression in each subgroup under each condition is different. *SoDof22*, *SoDof3,* and *SoDof15* showed the highest level in expression after treatment under cold, heat, and drought stress, respectively (Fig. 7B). As previous studies have shown, *Dof* genes participate in responding to various stresses. In tomato, *SlCDF1-5* genes were induced in response to osmotic, salt, heat, and low-temperature stresses. Over-expressing *SlCDF1* or *SlCDF3* in *Arabidopsis* showed an increasing drought and salt tolerance^55^. In brassica, the *BnCDF1* gene was induced in response to low temperatures, and overexpressing *BnCDF1* in Arabidopsis could increase freezing tolerance^56^. In watermelon, nine selected *Dof* genes showed differential expression under salt stress and ABA treatments^57^. In Chinese cabbage, most *Dof* genes were up-regulated quickly under salt, drought, heat and cold stresses^58^. Higher expression level of *SoDof22*, *SoDof3,* and *SoDof15* were detected after abiotic stress treatment, indicating that these genes might have an important role in responding to heat, cold and drought stresses. Over-expressing *BnCDF1* in *Arabidopsis* also delayed flowering time by reducing the expression of *CO* and *FT*^56^. *SoDof22* showed high expression level both in inflorescence and under cold stress, suggesting that the role of *SoDof22* might be similar to *BnCDF1* within the interplay between environmental conditions and flowering time.

The promoter of *SoDof22* contains an LTR *cis*-element responding to low-temperature and the promoter of *SoDof15* contains an MBS *cis*-element that participated in drought inducibility^59^ (Supplementary Data). The response of its *cis*-element leads to an increased expression under low temperature or PEG4000. According to the expression profile of each stress, there was an expression difference between each sex type in spinach. Under cold stress, *SoDof4* was down-regulated in female plants and *SoDof7* was down-regulated in female and vegetative plants. While, in male plants, they showed expression increase at 2 h after treatment. Under heat stress, *SoDof* genes in female plants were all up-regulated, while, vegetative plants and male plants contained down-regulated *SoDof* genes. Under drought stress, the quantity of down-regulated *SoDofs* in male plants was much more than that in others. Female plants are more sensitive to drought than male plants, similar to the response in *Populus yunnanensis*^60^.

## Supplementary Information

Supplementary Data.

Supplementary Figures.

Supplementary Tables.
