# Supplementary material for: Expression profiling of the Dof gene family under abiotic stresses in spinach
Source: Sci Rep. 2021 Jul 13;11:14429. doi: 10.1038/s41598-021-93383-6 (PMC8277872; doi:10.1038/s41598-021-93383-6)
Supplement: Supplementary file 2 — Supplementary Figures. [file 41598_2021_93383_MOESM2_ESM.docx]

Expression profiling of the Dof gene family under abiotic stresses in Spinach

Hongying Yu^1^, Yaying Ma^1^, Yijing Lu^1^, Jingjing Yue^2^ and Ray Ming^3*^

^1^College of Agriculture, Center for Genomics and Biotechnology, Fujian Provincial Key Laboratory of Haixia Applied Plant Systems Biology, Fujian Agriculture and Forestry University, Fuzhou, Fujian 350002, China.

^2^Center for Genomics and biotechnology, Fujian Agriculture and Forestry University, Fuzhou 350002, Fujian, China.

^3^Department of Plant Biology, University of Illinois at Urbana-Champaign, Urbana, IL 61801, USA.

*Corresponding author: rayming@illinois.edu

**Figure S1. The tissue-specific expression of all *Dof* genes in Spinach by qRT-PCR. The Y-axis indicates relative expression level and the X-axis indicated different tissues: root (gray); stem (light brown); leaf (green); female flower (red); male flower (pink). The error bars were calculated based on three biological replicates using standard deviation.**

**
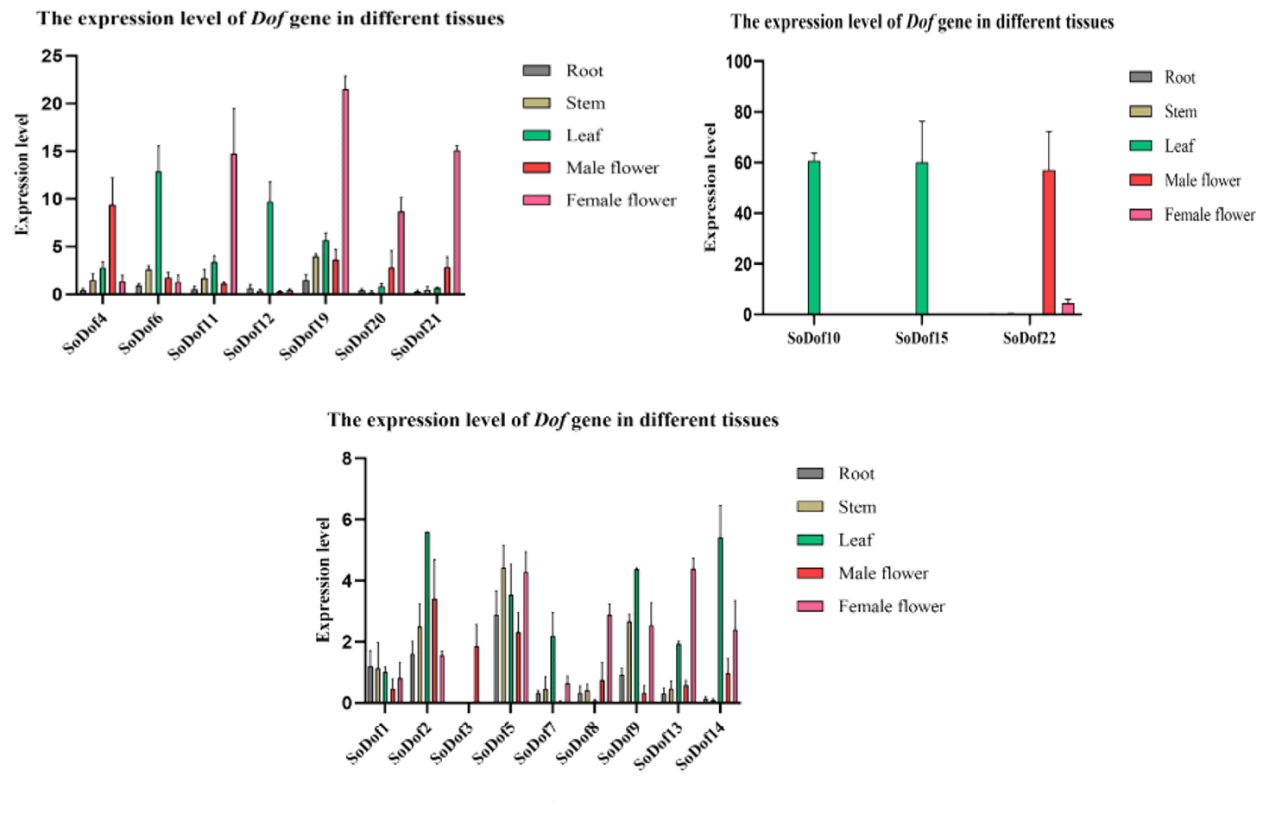
**

**Figure S2. The expression of all *Dof* genes under low temperature. The Y-axis indicates relative expression level and the X-axis indicated the time after treatment:0h (gray); 2h (light brown); 4h (orange); 7h (green); 12h (purple);24h (pink). *Asterisk* indicates a significant difference from 0h (*p*<0.05). *Error bars* indicate standard error of independent technological replicates. (A). The samples were collected in female plants. (B). The samples were collected in plant at vegetative stage. (C). The samples were collected in male plants.**

**A.**

**
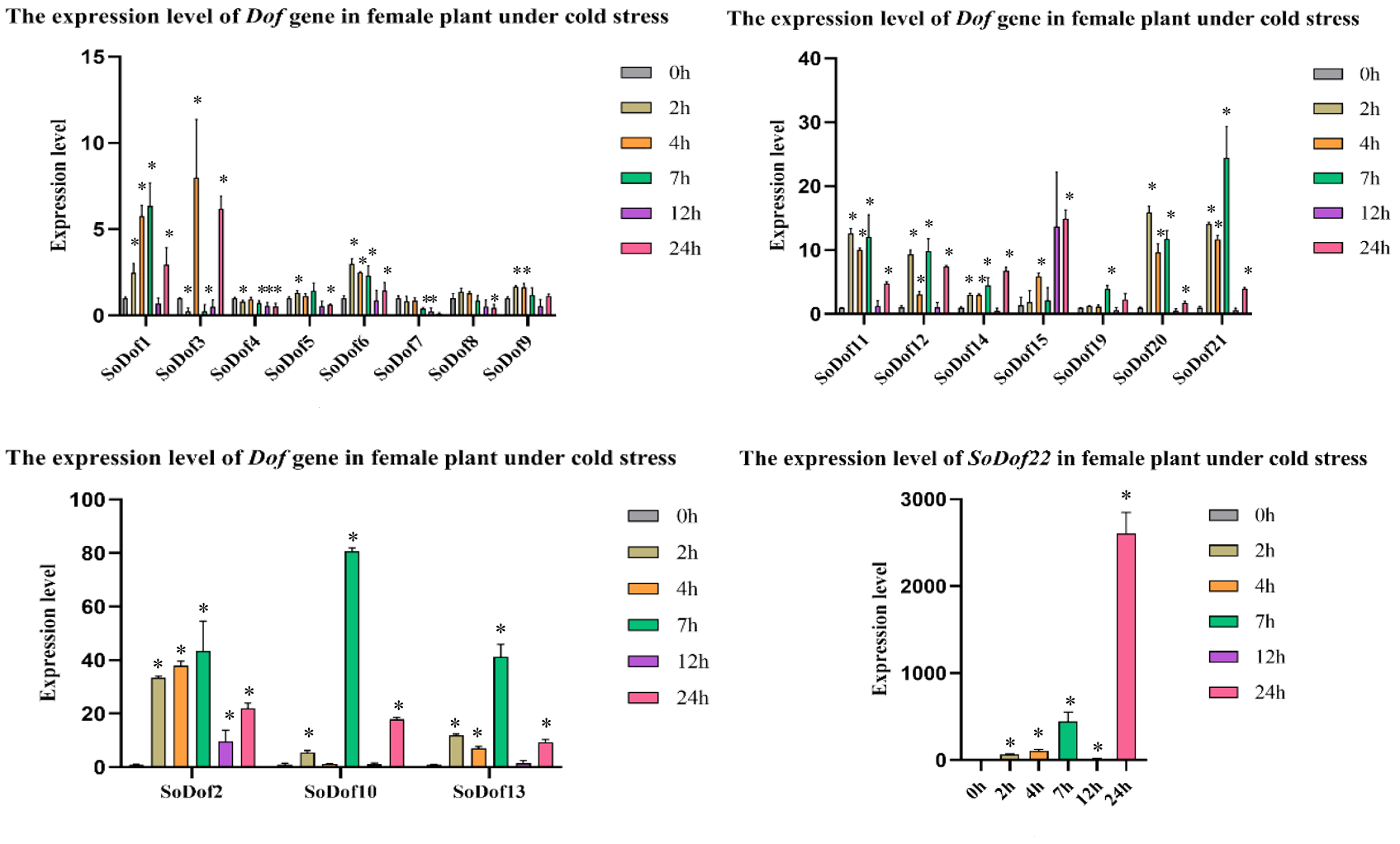
**

**B.**

**
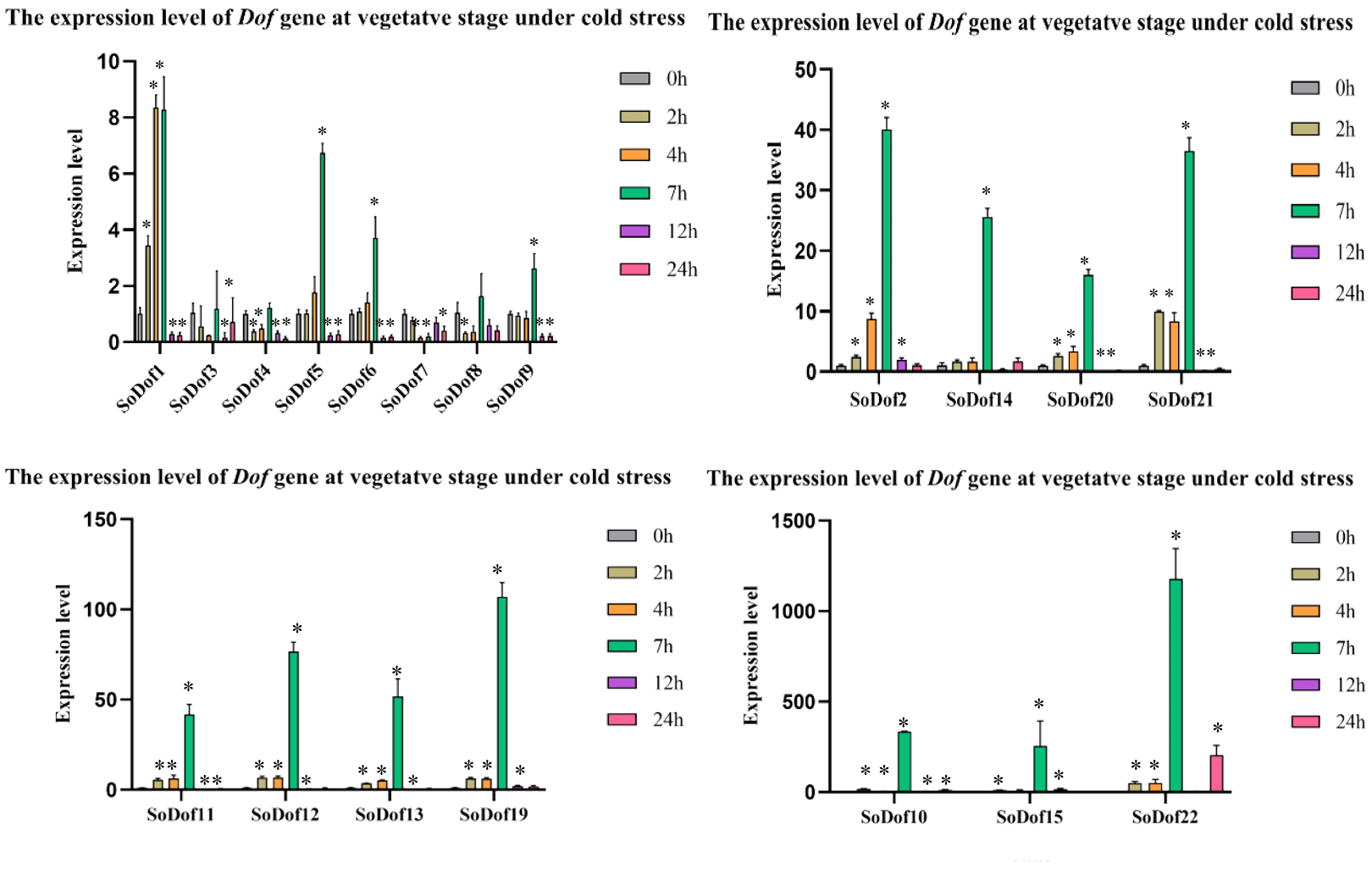
**

**C.**

**
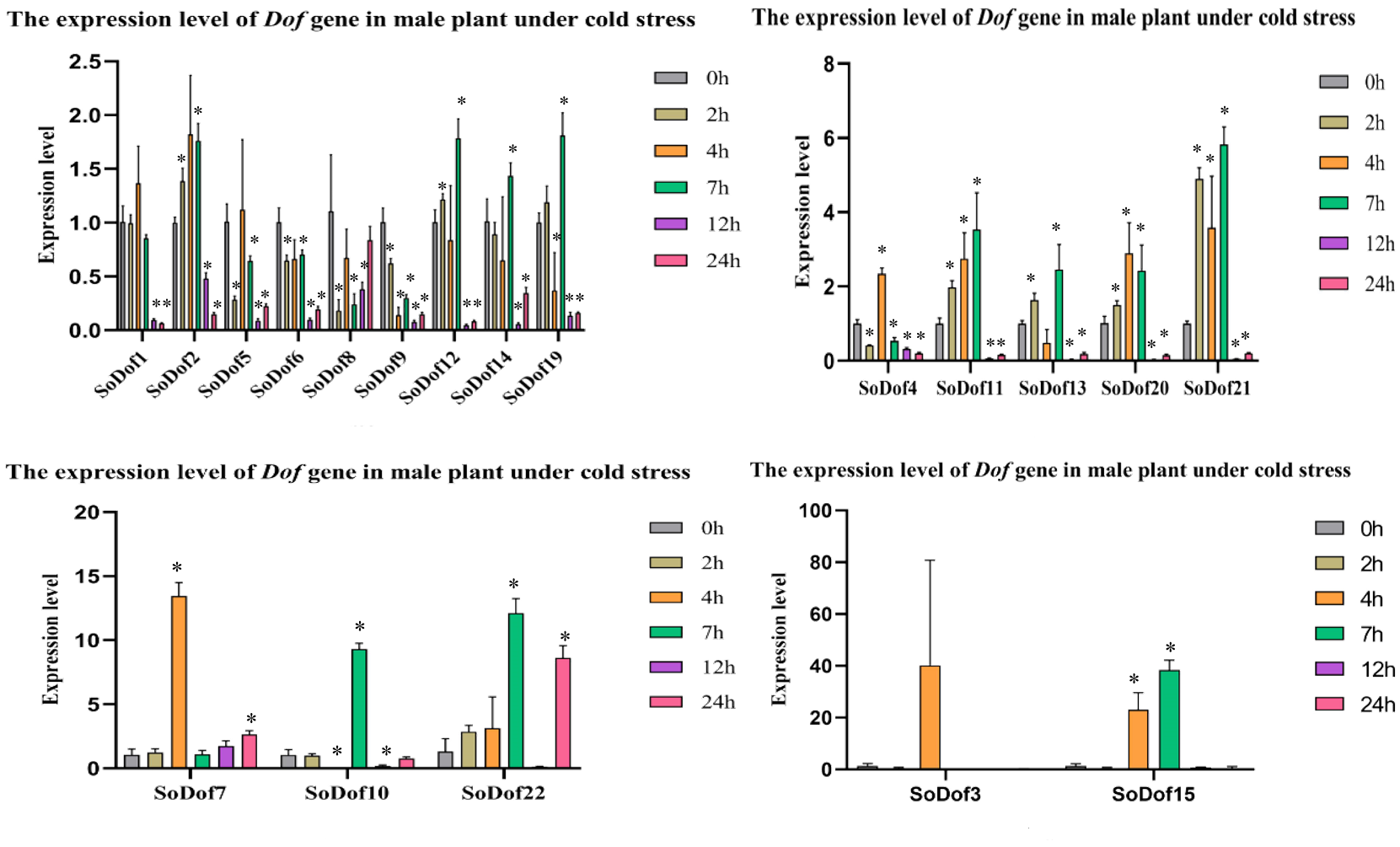
**

**Figure S3. The expression of all *Dof* genes under high temperature. The Y-axis indicates relative expression level and the X-axis indicated the time after treatment:0h (gray); 2h (light brown); 4h (orange); 7h (green); 12h (purple);24h (pink). *Asterisk* indicates a significant difference from 0h (*p*<0.05). *Error bars* indicate standard error of independent technological replicates. (B). The samples were collected in plant at vegetative stage. (C). The samples were collected in male plants.**

**A.**

**
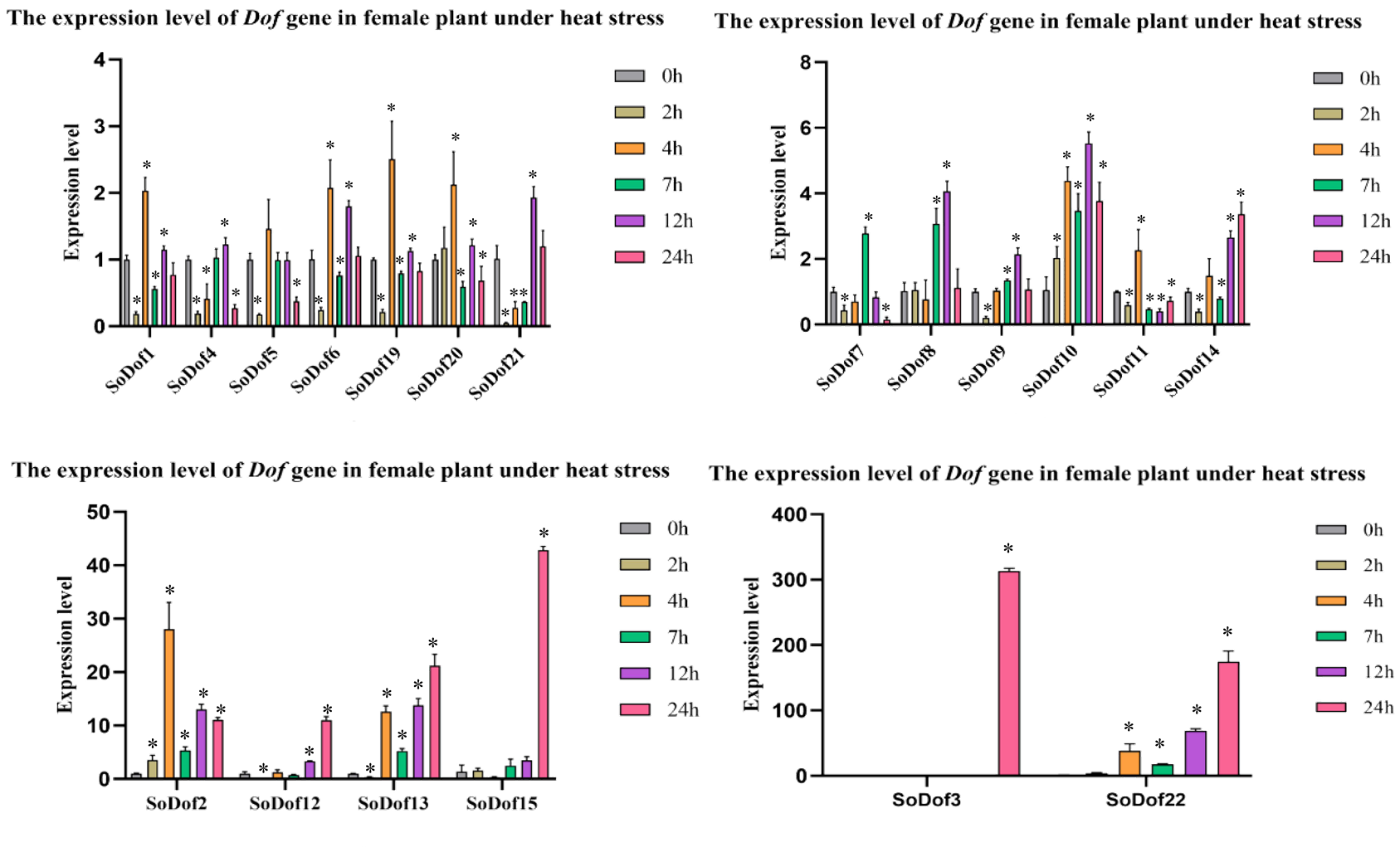
**

**B.**

**
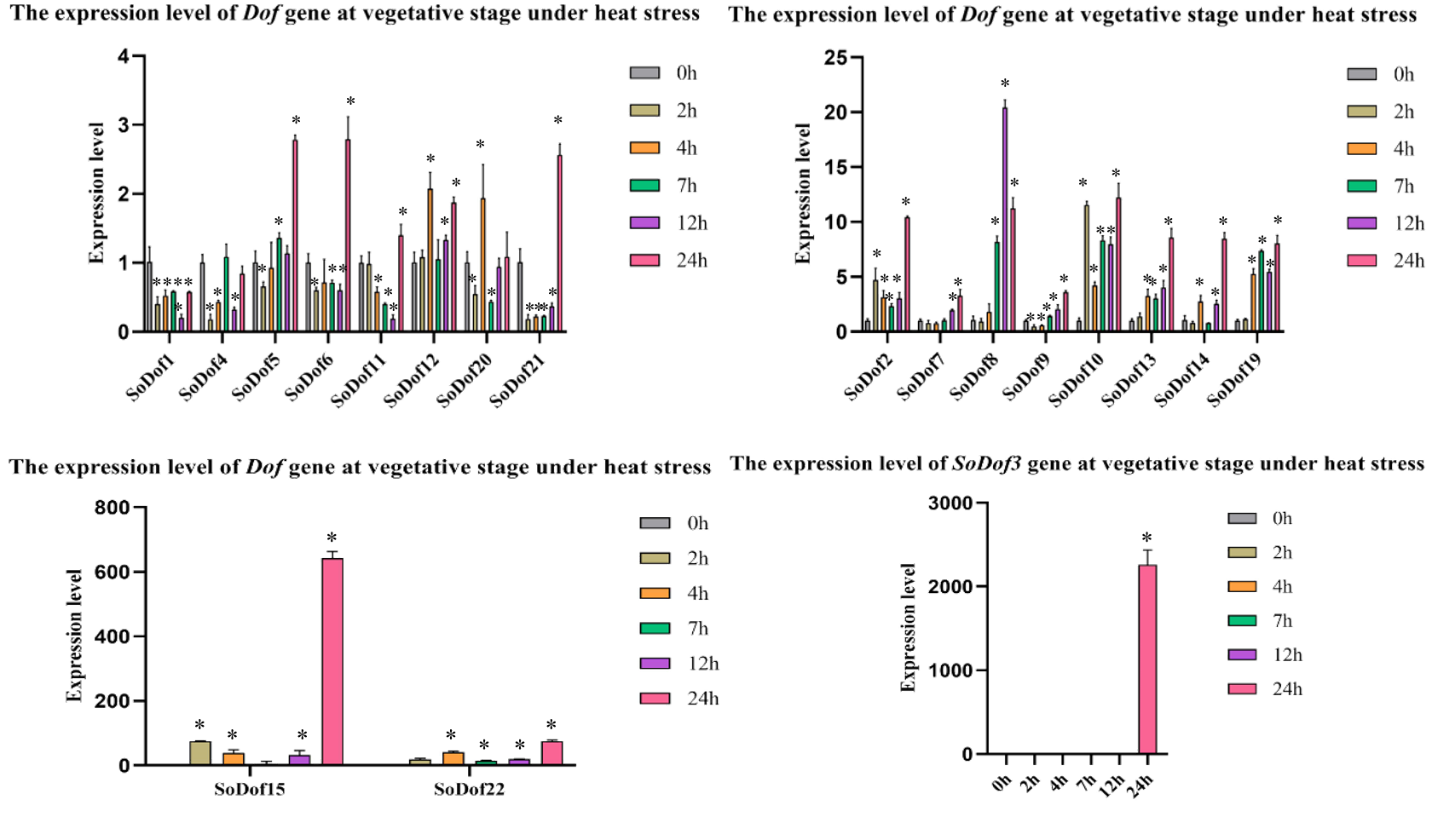
**

**C.**

**
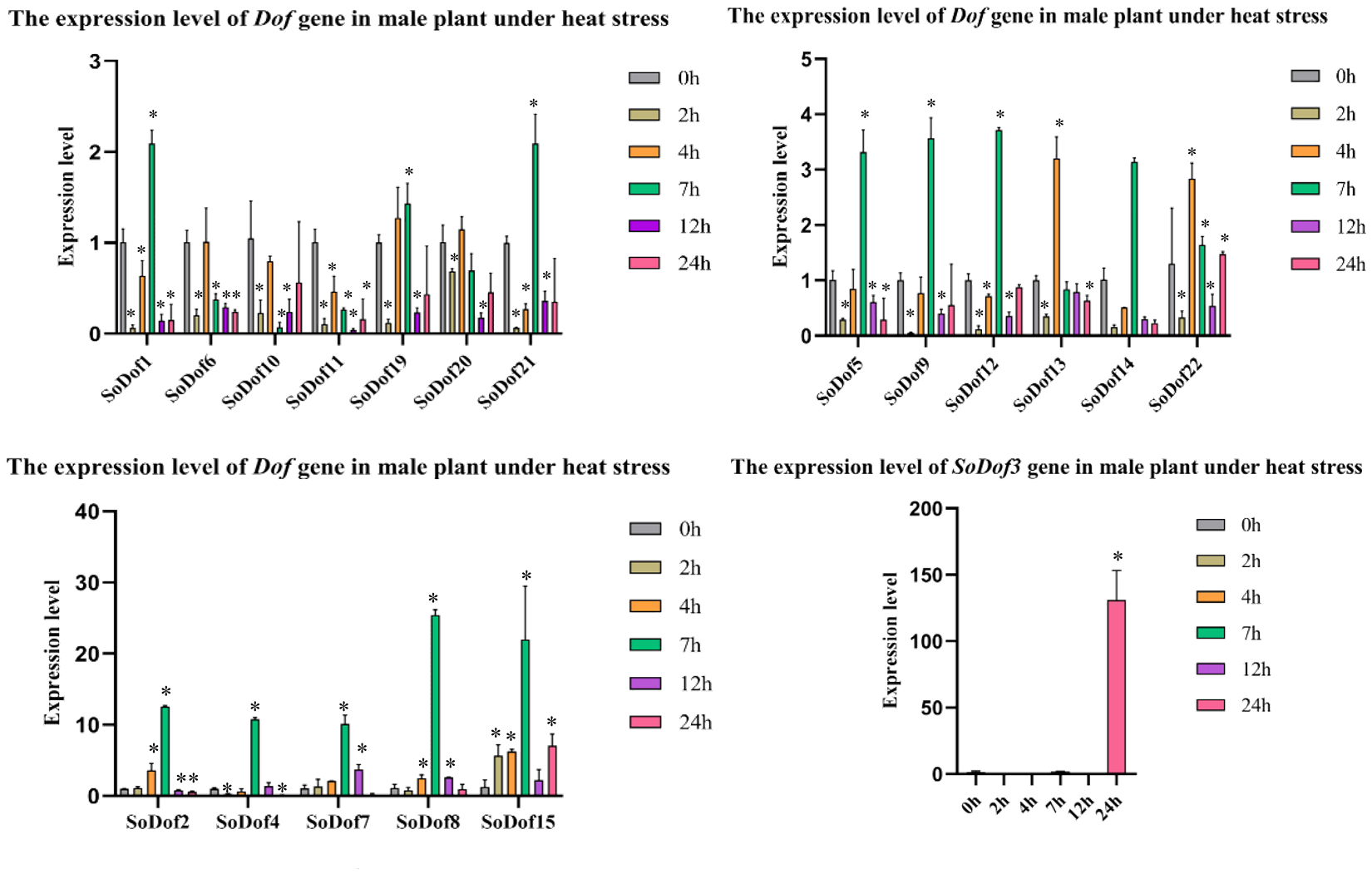
**

**Figure S4. The expression of all *Dof* genes under drought condition. The Y-axis indicates relative expression level and the X-axis indicated the time after treatment:0h (gray); 2h (light brown); 4h (orange); 7h (green); 12h (purple);24h (pink). *Asterisk* indicates a significant difference from 0h (*p*<0.05). *Error bars* indicate standard error of independent technological replicates. (A). The samples were collected in female plants. (B). The samples were collected in plant at vegetative stage. (C). The samples were collected in male plants.**

**A.**

**
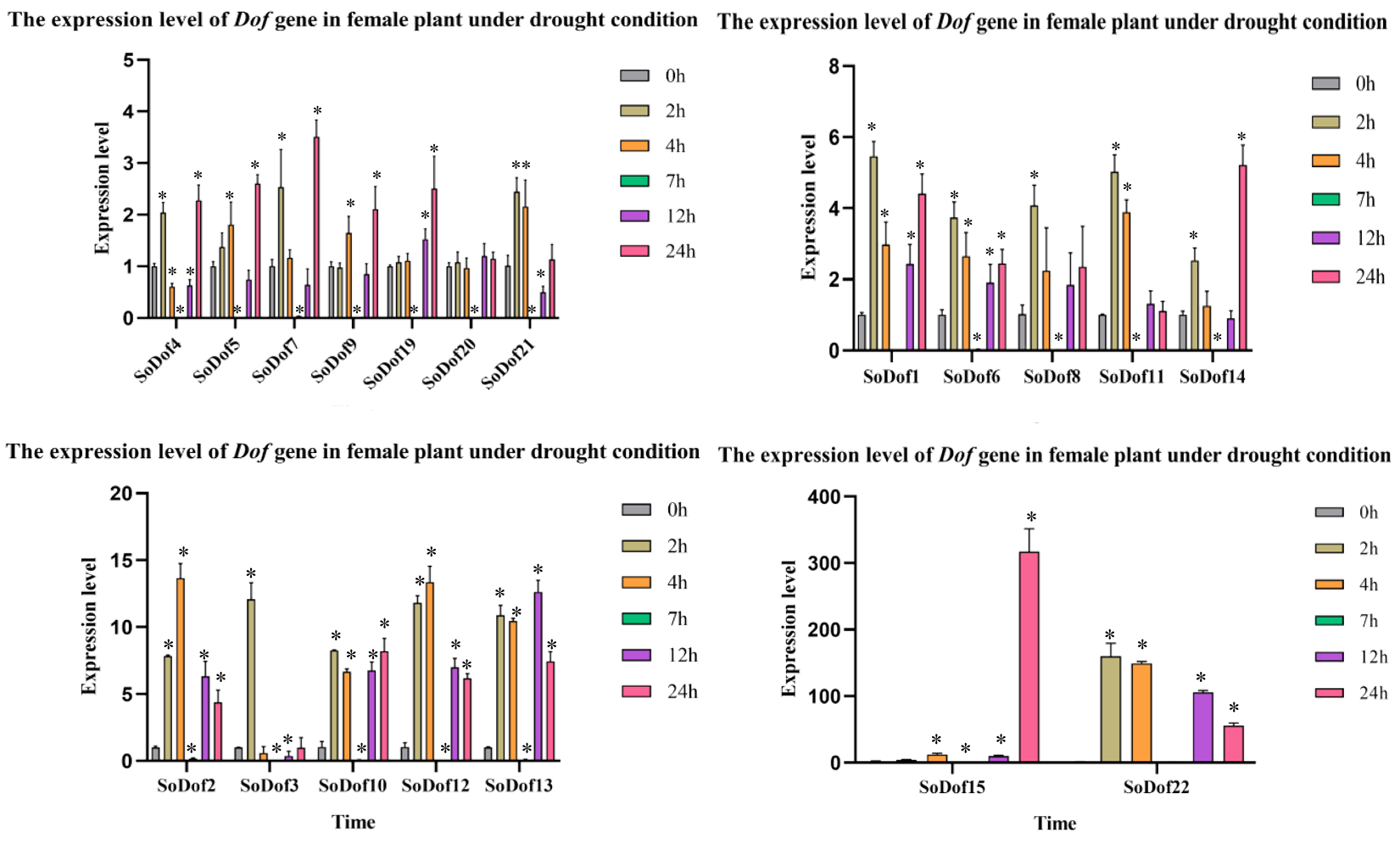
**

**B.**

**
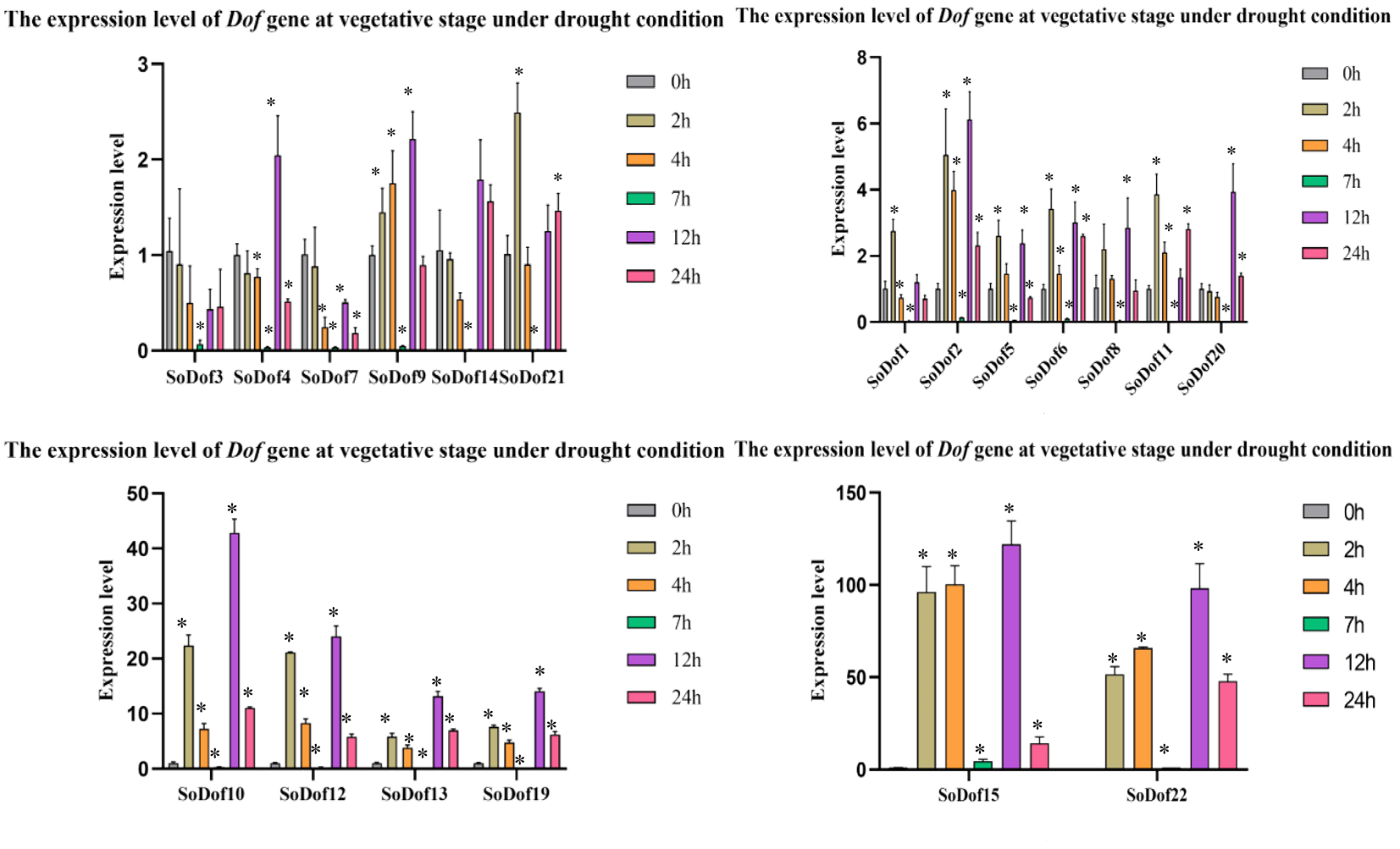
**

**C.**

**
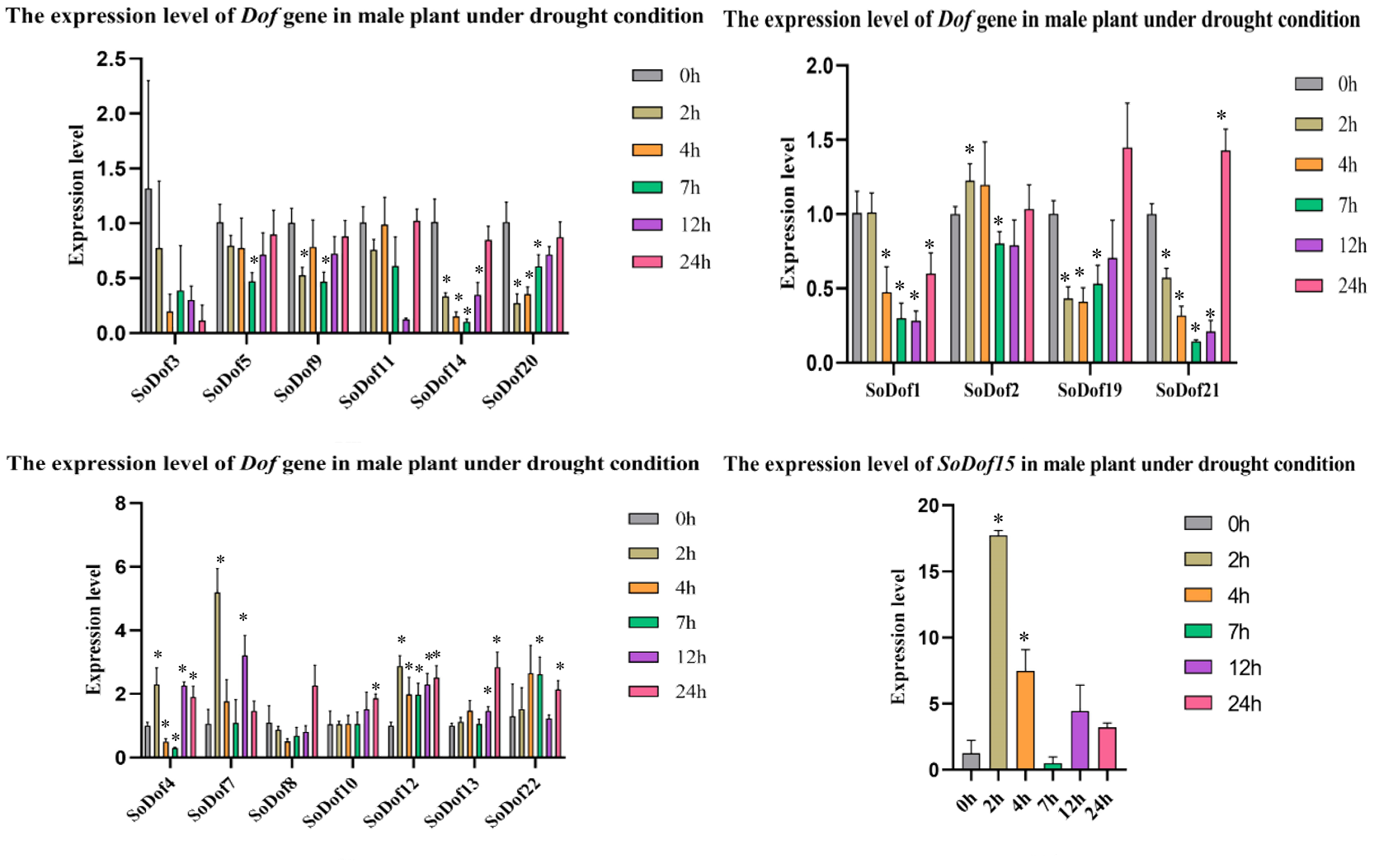
**
