# Supplementary material for: Expression profiling of the Dof gene family under abiotic stresses in spinach
Source: Sci Rep. 2021 Jul 13;11:14429. doi: 10.1038/s41598-021-93383-6 (PMC8277872; doi:10.1038/s41598-021-93383-6)
Supplement: Supplementary file 3 — Supplementary Tables. [file 41598_2021_93383_MOESM3_ESM.docx]

Expression profiling of the Dof gene family under abiotic stresses in Spinach

Hongying Yu^1^, Yaying Ma^1^, Yijing Lu^1^, Jingjing Yue^2^ and Ray Ming^3*^

^1^College of Agriculture, Center for Genomics and Biotechnology, Fujian Provincial Key Laboratory of Haixia Applied Plant Systems Biology, Fujian Agriculture and Forestry University, Fuzhou, Fujian 350002, China.

^2^Center for Genomics and biotechnology, Fujian Agriculture and Forestry University, Fuzhou 350002, Fujian, China.

^3^Department of Plant Biology, University of Illinois at Urbana-Champaign, Urbana, IL 61801, USA.

*Corresponding author: rayming@illinois.edu

**Table S1. *Dof* gene name of sugarbeet.**

| **Gene ID** | **Gene name** |
| --- | --- |
| KMT02174 | BvDof1 |
| KMT01677 | BvDof2 |
| KMT20267 | BvDof3 |
| KMT04631 | BvDof4 |
| KMT12430 | BvDof5 |
| KMT10595 | BvDof6 |
| KMT09301 | BvDof7 |
| KMT09375 | BvDof8 |
| KMT09384 | BvDof9 |
| KMT05269 | BvDof10 |
| KMT10808 | BvDof11 |
| KMT10807 | BvDof12 |
| KMT04634 | BvDof13 |
| KMT04410 | BvDof14 |
| KMS99249 | BvDof15 |
| KMT04296 | BvDof16 |
| KMT19467 | BvDof17 |
| KMT19469 | BvDof18 |
| KMT11536 | BvDof19 |
| KMT05268 | BvDof20 |
| KMT07044 | BvDof21 |
| KMS97626 | BvDof22 |

**Table S2. Ka/Ks analysis and estimated divergence time for *SoDof* pairs.**

| **Gene 1** | **Gene 2** | **Ks** | **Ka** | **Divergence time (mya)** | **Ka/Ks** |
| --- | --- | --- | --- | --- | --- |
| SoDof2 | SoDof3 | 5.2612 | 0.3741 | 37.58 | 0.071105451 |
| SoDof12 | SoDof21 | 3.7472 | 0.2989 | 26.76571429 | 0.079766225 |
| SoDof5 | SoDof15 | 4.1515 | 0.3321 | 29.65357143 | 0.079995182 |
| SoDof20 | SoDof22 | 5.7779 | 0.4813 | 41.27071429 | 0.083300161 |
| SoDof4 | SoDof7 | 5.2531 | 0.5222 | 37.52214286 | 0.099407969 |
| SoDof10 | SoDof20 | 4.706 | 0.4727 | 33.61428571 | 0.100446239 |
| SoDof13 | SoDof21 | 2.2537 | 0.2474 | 16.09785714 | 0.109775037 |
| SoDof2 | SoDof5 | 4.7018 | 0.5319 | 33.58428571 | 0.113126888 |
| SoDof6 | SoDof20 | 4.1988 | 0.4791 | 29.99142857 | 0.11410403 |
| SoDof6 | SoDof9 | 3.1391 | 0.4022 | 22.42214286 | 0.128125896 |
| SoDof7 | SoDof15 | 3.1922 | 0.4529 | 22.80142857 | 0.141877075 |
| SoDof1 | SoDof8 | 3.4678 | 0.5017 | 24.77 | 0.144673857 |
| SoDof11 | SoDof21 | 1.8648 | 0.2736 | 13.32 | 0.146718147 |
| SoDof14 | SoDof21 | 2.6953 | 0.3995 | 19.25214286 | 0.148220977 |
| SoDof3 | SoDof21 | 3.4168 | 0.5275 | 24.40571429 | 0.154384219 |
| SoDof4 | SoDof5 | 3.365 | 0.523 | 24.03571429 | 0.155423477 |
| SoDof8 | SoDof11 | 3.5994 | 0.5778 | 25.71 | 0.160526754 |
| SoDof6 | SoDof19 | 2.5298 | 0.4163 | 18.07 | 0.164558463 |
| SoDof8 | SoDof16 | 2.3407 | 0.3861 | 16.71928571 | 0.164950656 |
| SoDof8 | SoDof17 | 2.3407 | 0.3861 | 16.71928571 | 0.164950656 |
| SoDof8 | SoDof18 | 2.3407 | 0.3861 | 16.71928571 | 0.164950656 |
| SoDof4 | SoDof8 | 2.9279 | 0.4882 | 20.91357143 | 0.166740667 |
| SoDof6 | SoDof8 | 2.9112 | 0.4887 | 20.79428571 | 0.16786892 |
| SoDof8 | SoDof19 | 3.2036 | 0.5395 | 22.88285714 | 0.168404295 |
| SoDof5 | SoDof11 | 2.8869 | 0.5005 | 20.62071429 | 0.173369358 |
| SoDof11 | SoDof14 | 1.9352 | 0.348 | 13.82285714 | 0.179826375 |
| SoDof1 | SoDof22 | 2.6102 | 0.486 | 18.64428571 | 0.186192629 |
| SoDof3 | SoDof13 | 2.7251 | 0.5084 | 19.465 | 0.186561961 |
| SoDof5 | SoDof22 | 2.0462 | 0.3859 | 14.61571429 | 0.18859349 |
| SoDof10 | SoDof21 | 3.332 | 0.629 | 23.8 | 0.18877551 |
| SoDof6 | SoDof21 | 3.041 | 0.5948 | 21.72142857 | 0.195593555 |
| SoDof3 | SoDof9 | 2.3214 | 0.4567 | 16.58142857 | 0.196734729 |
| SoDof7 | SoDof8 | 2.0716 | 0.4089 | 14.79714286 | 0.197383665 |
| SoDof2 | SoDof11 | 2.9468 | 0.5849 | 21.04857143 | 0.198486494 |
| SoDof7 | SoDof10 | 2.3131 | 0.4685 | 16.52214286 | 0.202542043 |
| SoDof2 | SoDof8 | 1.9944 | 0.4111 | 14.24571429 | 0.206127156 |
| SoDof13 | SoDof14 | 1.7469 | 0.3614 | 12.47785714 | 0.20688076 |
| SoDof9 | SoDof10 | 2.0812 | 0.4327 | 14.86571429 | 0.207908899 |
| SoDof9 | SoDof15 | 1.8752 | 0.395 | 13.39428571 | 0.210644198 |
| SoDof5 | SoDof8 | 2.5755 | 0.5652 | 18.39642857 | 0.219452533 |
| SoDof4 | SoDof22 | 2.4843 | 0.5496 | 17.745 | 0.22122932 |
| SoDof1 | SoDof5 | 1.1508 | 0.2573 | 8.22 | 0.223583594 |
| SoDof5 | SoDof9 | 2.2965 | 0.5155 | 16.40357143 | 0.224472023 |
| SoDof9 | SoDof22 | 2.4402 | 0.5583 | 17.43 | 0.228792722 |
| SoDof2 | SoDof4 | 1.7764 | 0.4118 | 12.68857143 | 0.231817158 |
| SoDof1 | SoDof10 | 1.7287 | 0.4054 | 12.34785714 | 0.234511483 |
| SoDof11 | SoDof22 | 2.6918 | 0.6355 | 19.22714286 | 0.236087376 |
| SoDof5 | SoDof16 | 2.3671 | 0.5665 | 16.90785714 | 0.239322378 |
| SoDof5 | SoDof17 | 2.3671 | 0.5665 | 16.90785714 | 0.239322378 |
| SoDof5 | SoDof18 | 2.3671 | 0.5665 | 16.90785714 | 0.239322378 |
| SoDof3 | SoDof22 | 2.1195 | 0.5082 | 15.13928571 | 0.239773531 |
| SoDof10 | SoDof16 | 1.4251 | 0.3493 | 10.17928571 | 0.245105607 |
| SoDof10 | SoDof17 | 1.4251 | 0.3493 | 10.17928571 | 0.245105607 |
| SoDof10 | SoDof18 | 1.4251 | 0.3493 | 10.17928571 | 0.245105607 |
| SoDof16 | SoDof20 | 1.7815 | 0.4503 | 12.725 | 0.252764524 |
| SoDof17 | SoDof20 | 1.7815 | 0.4503 | 12.725 | 0.252764524 |
| SoDof18 | SoDof20 | 1.7815 | 0.4503 | 12.725 | 0.252764524 |
| SoDof8 | SoDof20 | 1.9111 | 0.4943 | 13.65071429 | 0.258646853 |
| SoDof5 | SoDof21 | 2.1025 | 0.5496 | 15.01785714 | 0.261403092 |
| SoDof9 | SoDof20 | 1.9969 | 0.5246 | 14.26357143 | 0.262707196 |
| SoDof6 | SoDof7 | 1.8018 | 0.4749 | 12.87 | 0.263569764 |
| SoDof5 | SoDof14 | 1.9222 | 0.5315 | 13.73 | 0.276506087 |
| SoDof5 | SoDof20 | 1.6264 | 0.45 | 11.61714286 | 0.276684702 |
| SoDof12 | SoDof16 | 2.4483 | 0.6821 | 17.48785714 | 0.278601479 |
| SoDof12 | SoDof17 | 2.4483 | 0.6821 | 17.48785714 | 0.278601479 |
| SoDof12 | SoDof18 | 2.4483 | 0.6821 | 17.48785714 | 0.278601479 |
| SoDof8 | SoDof9 | 1.943 | 0.5535 | 13.87857143 | 0.28486876 |
| SoDof10 | SoDof15 | 1.7898 | 0.5137 | 12.78428571 | 0.287015309 |
| SoDof2 | SoDof12 | 2.2753 | 0.6555 | 16.25214286 | 0.288093878 |
| SoDof1 | SoDof15 | 1.3555 | 0.4022 | 9.682142857 | 0.296717079 |
| SoDof20 | SoDof21 | 2.254 | 0.6776 | 16.1 | 0.300621118 |
| SoDof3 | SoDof8 | 1.4802 | 0.4467 | 10.57285714 | 0.301783543 |
| SoDof10 | SoDof12 | 1.9382 | 0.588 | 13.84428571 | 0.303374265 |
| SoDof7 | SoDof22 | 1.7458 | 0.5321 | 12.47 | 0.304788636 |
| SoDof5 | SoDof19 | 1.7591 | 0.5421 | 12.565 | 0.30816895 |
| SoDof15 | SoDof19 | 1.551 | 0.4803 | 11.07857143 | 0.30967118 |
| SoDof4 | SoDof14 | 2.3587 | 0.7341 | 16.84785714 | 0.311230763 |
| SoDof14 | SoDof19 | 2.115 | 0.6711 | 15.10714286 | 0.317304965 |
| SoDof2 | SoDof9 | 1.4121 | 0.4607 | 10.08642857 | 0.326251682 |
| SoDof15 | SoDof21 | 1.797 | 0.587 | 12.83571429 | 0.326655537 |
| SoDof1 | SoDof9 | 1.6562 | 0.544 | 11.83 | 0.328462746 |
| SoDof5 | SoDof7 | 1.4937 | 0.4927 | 10.66928571 | 0.329852045 |
| SoDof1 | SoDof7 | 1.5349 | 0.5101 | 10.96357143 | 0.332334354 |
| SoDof19 | SoDof22 | 1.8752 | 0.6305 | 13.39428571 | 0.336230802 |
| SoDof8 | SoDof21 | 1.8213 | 0.6129 | 13.00928571 | 0.336517872 |
| SoDof7 | SoDof9 | 1.3438 | 0.4677 | 9.598571429 | 0.348042864 |
| SoDof1 | SoDof19 | 1.6608 | 0.5915 | 11.86285714 | 0.356153661 |
| SoDof3 | SoDof20 | 1.3376 | 0.4816 | 9.554285714 | 0.360047847 |
| SoDof1 | SoDof6 | 1.3101 | 0.4862 | 9.357857143 | 0.371116709 |
| SoDof16 | SoDof22 | 1.6363 | 0.6229 | 11.68785714 | 0.380675915 |
| SoDof17 | SoDof22 | 1.6363 | 0.6229 | 11.68785714 | 0.380675915 |
| SoDof18 | SoDof22 | 1.6363 | 0.6229 | 11.68785714 | 0.380675915 |
| SoDof5 | SoDof6 | 1.1712 | 0.4524 | 8.365714286 | 0.386270492 |
| SoDof10 | SoDof13 | 1.6565 | 0.661 | 11.83214286 | 0.399034108 |
| SoDof6 | SoDof13 | 1.4366 | 0.5755 | 10.26142857 | 0.400598636 |
| SoDof7 | SoDof19 | 1.1 | 0.481 | 7.857142857 | 0.437272727 |
| SoDof4 | SoDof12 | 1.4111 | 0.634 | 10.07928571 | 0.449294876 |
| SoDof4 | SoDof11 | 1.4412 | 0.6672 | 10.29428571 | 0.462947544 |
| SoDof6 | SoDof11 | 1.1265 | 0.539 | 8.046428571 | 0.478473147 |
| SoDof11 | SoDof16 | 1.2138 | 0.6644 | 8.67 | 0.54737189 |
| SoDof11 | SoDof17 | 1.2138 | 0.6644 | 8.67 | 0.54737189 |
| SoDof11 | SoDof18 | 1.2138 | 0.6644 | 8.67 | 0.54737189 |
| SoDof9 | SoDof19 | 0.793 | 0.5134 | 5.664285714 | 0.64741488 |

**Table S3. qRT-PCR primers used in this study.**

| **Gene name** | **Sequence of primer (5'-3')** | |
| --- | --- | --- |
|  | **F** | **R** |
| SoDof1 | GATGATCAAAGCAAAGAAGGAACA | CACCTAGCATACCACTCCAATAC |
| SoDof2 | GAGGGACAATTCGGTTCCTTAT | CCACTCCCATGAAGCAGTT |
| SoDof3 | CTGGAACTGGTGGGTGTTT | GATGACGATGATGAAGAGGAGTT |
| SoDof4 | GGCCACCATGAGGAAGATAA | CCCTGTTCTGCAACAAATCC |
| SoDof5 | TGGGTTTCCGCTACAAGATTAC | GTTGATGATGGTGGTGCATTTC |
| SoDof6 | GCTACTACAACTTCACCCACAA | CATCTTCGTACGAGGGCAATAG |
| SoDof7 | GGAGGAGGAGGAGGAAATAGA | CCTCCTATTCCACCCATAATACC |
| SoDof8 | AGAAGGAGGTGGTGTGAGTA | GCAAGGTCTGGTAAGGATGTAA |
| SoDof9 | CGGATCTCCATTCAGCTTCTT | TAGGGTTAGGGTTAGGGTTAGG |
| SoDof10 | TCTCTCCTCTCCAACCAGAAT | GGGAAGCCCGGTGAAATTA |
| SoDof11 | ACGAATCAAATGCCGTGTTATC | CCAATAAGGAGGCGGGTAAA |
| SoDof12 | TGGATGGTATGGTTGTGGATG | CAGTAGACTCACTCCTCCTTCT |
| SoDof13 | TTCCCGAAGCACTCCATAATC | GGGTGTATCTGAGCTGAAAGTAA |
| SoDof14 | TCAGATTGTTCCTGCTGCTTAT | GATGGAGAGACACACCCATTT |
| SoDof15 | GATAGACCTCGCGGTTGTTTA | ACCACCACTAGGGAGTACAT |
| SoDof19 | GCTTCCAAACCCTCCTTCTT | CAGTTGCTCAGGTTGTTGTTG |
| SoDof20 | ACCCTGATTTGGCCTCTTTC | ATACCGGCTGCCATGTAAAG |
| SoDof21 | GTTAGCTCTTCAATCGGGAGAATA | GGATTGGCTTGTAGAACCATAGA |
| SoDof22 | CTGCTGCTCTGTCGTCAATAA | ATCGTCGTTGTAGATTGGTTCC |
| actin-11 | CGAGCTGTGTTCCCTAGTATTG | CAAGATTCCACGCTTTGATTGAG |
